# Deciphering Signaling Pathway Networks to Understand the Molecular Mechanisms of Metformin Action

**DOI:** 10.1371/journal.pcbi.1004202

**Published:** 2015-06-17

**Authors:** Jingchun Sun, Min Zhao, Peilin Jia, Lily Wang, Yonghui Wu, Carissa Iverson, Yubo Zhou, Erica Bowton, Dan M. Roden, Joshua C. Denny, Melinda C. Aldrich, Hua Xu, Zhongming Zhao

**Affiliations:** 1 School of Biomedical Informatics, The University of Texas Health Science Center at Houston, Houston, Texas, United States of America; 2 Department of Biomedical Informatics, Vanderbilt University School of Medicine, Nashville, Tennessee, United States of America; 3 Department of Biostatistics, Vanderbilt University School of Medicine, Nashville, Tennessee, United States of America; 4 Department of Thoracic Surgery, Vanderbilt University School of Medicine, Nashville, Tennessee, United States of America; 5 Center for Human Genetics Research, Vanderbilt University, Nashville, Tennessee, United States of America; 6 National Center for Drug Screening, Shanghai Institute of Materia Medica, Chinese Academy of Sciences, Shanghai, People’s Republic of China; 7 Institute for Clinical and Translational Research, School of Medicine, Vanderbilt University, Nashville, Tennessee, United States of America; 8 Department of Medicine, Vanderbilt University School of Medicine, Nashville, Tennessee, United States of America; 9 Department of Pharmacology, Vanderbilt University School of Medicine, Nashville, Tennessee, United States of America; 10 Division of Epidemiology, Vanderbilt University School of Medicine, Nashville, Tennessee, United States of America; 11 Center for Quantitative Sciences, Vanderbilt University School of Medicine, Nashville, Tennessee, United States of America; 12 Department of Cancer Biology, Vanderbilt University School of Medicine, Nashville, Tennessee, United States of America; University of Southern California, UNITED STATES

## Abstract

A drug exerts its effects typically through a signal transduction cascade, which is non-linear and involves intertwined networks of multiple signaling pathways. Construction of such a signaling pathway network (SPNetwork) can enable identification of novel drug targets and deep understanding of drug action. However, it is challenging to synopsize critical components of these interwoven pathways into one network. To tackle this issue, we developed a novel computational framework, the Drug-specific Signaling Pathway Network (DSPathNet). The DSPathNet amalgamates the prior drug knowledge and drug-induced gene expression via random walk algorithms. Using the drug metformin, we illustrated this framework and obtained one metformin-specific SPNetwork containing 477 nodes and 1,366 edges. To evaluate this network, we performed the gene set enrichment analysis using the disease genes of type 2 diabetes (T2D) and cancer, one T2D genome-wide association study (GWAS) dataset, three cancer GWAS datasets, and one GWAS dataset of cancer patients with T2D on metformin. The results showed that the metformin network was significantly enriched with disease genes for both T2D and cancer, and that the network also included genes that may be associated with metformin-associated cancer survival. Furthermore, from the metformin SPNetwork and common genes to T2D and cancer, we generated a subnetwork to highlight the molecule crosstalk between T2D and cancer. The follow-up network analyses and literature mining revealed that seven genes (*CDKN1A*, *ESR1*, *MAX*, *MYC*, *PPARGC1A*, *SP1*, and *STK11*) and one novel MYC-centered pathway with *CDKN1A*, *SP1*, and *STK11* might play important roles in metformin’s antidiabetic and anticancer effects. Some results are supported by previous studies. In summary, our study 1) develops a novel framework to construct drug-specific signal transduction networks; 2) provides insights into the molecular mode of metformin; 3) serves a model for exploring signaling pathways to facilitate understanding of drug action, disease pathogenesis, and identification of drug targets.

## Author Summary

A deep understanding of a drug’s mechanisms of actions is essential not only in the discovery of new treatments but also in minimizing adverse effects. Here, we develop a computational framework, the Drug-specific Signaling Pathway Network (DSPathNet), to reconstruct a comprehensive signaling pathway network (SPNetwork) impacted by a particular drug. To illustrate this computational approach, we used metformin, an anti-diabetic drug, as an example. Starting from collecting the metformin-related upstream genes and inferring the metformin-related downstream genes, we built one metformin-specific SPNetwork via random walk based algorithms. Our evaluation of the metformin-specific SPNetwork by using disease genes and genotyping data from genome-wide association studies showed that our DSPathNet approach was efficient to synopsize drug’s key components and their relationship involved in the type 2 diabetes and cancer, even the metformin anticancer activity. This work presents a novel computational framework for constructing individual drug-specific signal transduction networks. Furthermore, its successful application to the drug metformin provides some valuable insights into the mode of metformin action, which will facilitate our understanding of the molecular mechanisms underlying drug treatments, disease pathogenesis, and identification of novel drug targets and repurposed drugs.

## Introduction

Most drugs exert their therapeutic actions through interactions with specific protein targets. These target proteins are dominated by two categories: enzymes that catalyze reactions essential for the functioning of organisms, and receptors that transmit signals by interacting with messenger molecules [1,2]. The interactions of drugs and their targets initiate the signal transduction cascade that is usually propagated by the involved proteins and multiple pathways. These proteins and pathways act in the mode of crosstalk networks [3]. The process of such signaling transduction converts the chemical signals to a specific cellular response such as gene expression, cell division, and inhibition of cell death and apoptosis [4]. The signaling cascade usually ends at the recipients of chemical signals such as transcription factors (TFs), which have specific binding sites on DNA and play critical roles in the gene expression regulation [5]. In complex diseases such as cancer [6,7], neuropsychiatric disorders [8], and diabetes [9], these molecules involved in the signal transduction cascade that are altered and, thus, become attractive targets for disease treatment [10,11]. Therefore, targeting signaling pathways has become an important approach to discovering new drugs through traditional experimental methods [12,13] and to predicting drug repositioning through systematic approaches [14]. However, the primary challenge for utilizing signal transduction pathways for drug discovery is to synopsize the drug signaling pathways into one comprehensive system, including the major causal genetic factors for pathology of the complex disease and the most elemental components in the drug action.

Recent high-throughput technologies such as array-based mRNA and microRNA expression, genome-wide association studies (GWAS), and next-generation sequencing (NGS) have provided massive amounts of data, enabling investigation of drug effect through pharmacogenomic network approaches. For example, the Connectivity Map (CMap, build 02) studied the effect of 1,309 small chemicals on gene expression in four cultured human cells [15]. Furthermore, multiple reliable drug-centered databases such as DrugBank [16], KEGG (Kyoto Encyclopedia of Genes and Genomes) DRUG [17], PharmGKB (The Pharmacogenomics Knowledge Base) [18], and STITCH (Search Tool for Interactions Chemicals) [19], provide comprehensive and detailed drug information for computational discovery and/or drug design. Therefore, it is possible to integrate known drug targets, genes involved in drug pharmacokinetics (PK) and pharmacodynamics (PD) processes, drug-induced gene expression data, and disease-gene associations. Additionally, network-assisted approaches have become powerful tools to explore disease-gene, gene-gene, as well as drug-target associations in pharmacology and human disease [20–23]. Therefore, we hypothesized that the construction of a signaling pathway network to connect the upstream components and downstream signal recipients for an individual drug would increase power to identify genes that play critical roles in drug action or disease development.

In this study, we develop a computational framework, called DSPathNet, to construct one signaling pathway network (SPNetwork) for a particular drug via amalgamating drug knowledge with drug-induced gene expression data. The main purposes are to capture the principal components in the drug signal transduction process and to provide an alternative approach to identifying critical elements and modules (subnetworks) relevant to drug action. We illustrate the utility of DSPathNet using the metformin, one of the most widely prescribed anti-diabetic drugs in the world which has been recently shown to be useful for cancer treatment and prevention in people at higher risk [24–26]. We started with the collection of known drug-related genes and inference of TFs from metformin-induced gene expression data. Considering that most of the known drug-related genes participate in PK and PD processes and are located in the upstream of the signaling cascade based on their function, we defined them as “metformin upstream genes.” Likewise, we defined the TFs that receive and transmit the chemical signals at the end of the signaling cascade as “metformin downstream genes.” After overlaying the two sets of genes onto human SPNetwork, we employed random walk algorithms to construct a metformin-specific SPNetwork. The random walk-based methodology aims to identify the pathways that are closet to the known disease genes compared to other methods [27] and offers the best predictive performance [28]. The network is expected to enrich with signaling genes involved in metformin signal transduction. We performed the comprehensive gene enrichment analyses of the network using the disease genes of type 2 diabetes (T2D) from GWAS catalog [29], cancer genes from Cancer Gene Census [30], one T2D GWAS [31], three cancer GWAS [32,33], and one novel GWAS of cancer patients with T2D using metformin from BioVU [34]. The enrichment analysis results showed that the network contained a significant number of T2D and cancer disease genes and genes related to metformin action, indicating that the framework is promising as a method to identify critical genes involved in disease pathology and drug action. Additionally, the metformin-specific SPNetwork generated here provides potential metformin targets and molecular insights for further delineating the mechanism of metformin action.

## Results

### DSPathNet, a novel computational framework for exploring drug-specific signaling pathway network

In this study, we develop a novel computational framework to build a *D*rug-specific *S*ignaling *Path*way *Net*work, namely DSPathNet, for constructing a signaling pathway network (SPNetwork) for an individual drug of interest. The drug-specific SPNetwork is expected to contain critical components in the drug’s signal transduction cascade. These components are genes that harbor genetic variations contributing to the pathology of the drug indication or drug response. Thus, the drug-specific SPNetwork would facilitate our understanding of the molecular mechanisms of drug action, disease pathogenesis, and identification of novel drug targets. To prove the principle, we utilized the drug metformin as an example to evaluate the framework.

**Fig 1:**
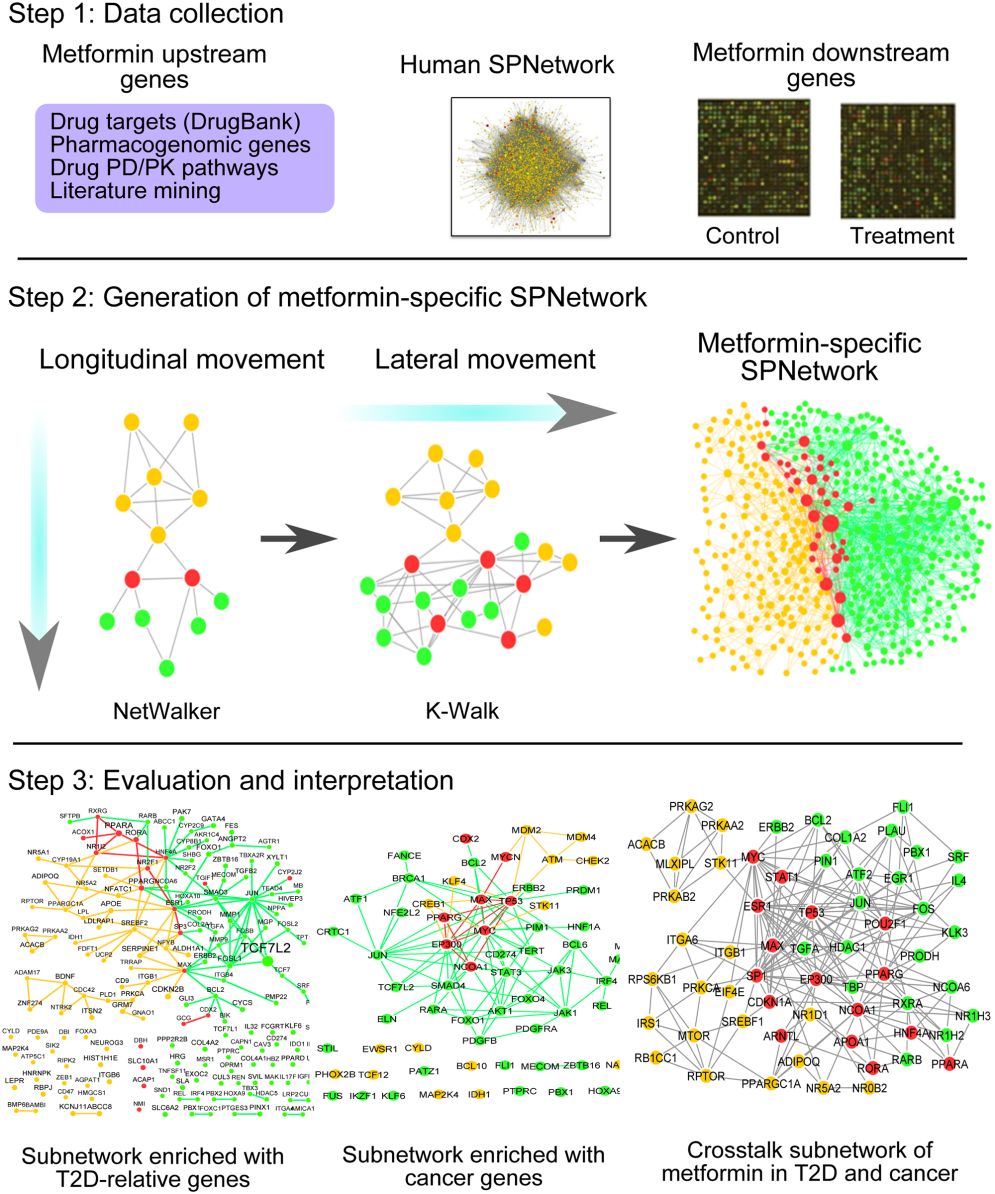
Overview of DSPathNet, a novel computational framework to construct a drug-specific signaling pathway network (SPNetwork): metformin as a case. Step 1: we collected the metformin upstream genes from multiple sources and inferred metformin downstream genes from metformin-induced gene expression data. We also compiled one human SPNetwork. Step 2: we utilized the metformin upstream and downstream genes as seeds to generate a metformin-specific SPNetwork from the human SPNetwork. The process involved longitudinal and lateral movements. Step 3: we utilized disease genes and genome-wide association studies (GWAS) data to evaluate if the metformin-specific SPNetwork was enriched with disease genes for type 2 diabetes (T2D) and cancer, genes associated with metformin action. Furthermore, we derived a crosstalk network of metformin action for T2D and cancer in order to identify key components in the metformin signal transduction via network topological and functional analysis. The nodes in orange correspond to the drug-related upstream genes, the nodes in green to the drug-related downstream genes, and the nodes in red to the nodes common to the upstream and downstream gene networks.

Fig 1 outlines the framework to build the metformin-specific SPNetwork and S1 Table summarizes the data sources, software and evaluation data used in the study. Briefly, we first collected metformin upstream genes from multiple sources and inferred metformin downstream genes from metformin-induced gene expression data. We compiled a human SPNetwork from two databases, Pathway Commons [35] and TRANSFAC [36], as a background pathway system for all signal transduction processes in humans. To weight the association of each node with metformin action, we assigned a functional similarity score to each node based on their Gene Ontology (GO) annotations and metformin upstream genes. The human SPNetwork included 37,881 edges and 4,367 nodes. Then, we utilized the metformin upstream and downstream genes as seeds to produce the metformin-specific SPNetwork from the human SPNetwork via random walk approaches. In this process, we applied a crossing network strategy to generate the drug-specific SPNetwork from background human SPNetwork by longitudinal and lateral movements. Finally, we computationally evaluated the metformin-specific SPNetwork by examining the enrichment of genes in the network using two types of data. The first includes the disease genes of type 2 diabetes (T2D) and cancer, the two diseases in which metformin has been actively studied. The second contains the individual genotyping data from five GWAS datasets: one T2D GWAS dataset, three cancer GWAS datasets, and one GWAS dataset of cancer patients with T2D treated by metformin. Our evaluation results indicated that the metformin-specific SPNetwork was significantly enriched with genes with mutations that could contribute to the pathology of T2D and cancer, and genes that may be associated with metformin-associated cancer survival (Table 1). To further investigate the molecular mechanisms underlying metformin action, we built a crosstalk subnetwork based on common genes to T2D and cancer, network topology, and functional analyses. We revealed several critical components, modules, and pathways that might be involved in metformin action.

**Table 1:** Comparison of genes in metformin-specific signaling pathway network with T2D and cancer genes and genes with smallest P-value less than 0.05 in five GWAS data sets.

Data	Number of genes^a^	Number of genes with smallest *P*< 0.05	Hypergeometric test *P*-value^b^
T2D disease genes	131	11	1.36 × 10^–4^
T2D GWAS	445	169	3.08 × 10^–5^
Cancer genes	509	64	1.64 × 10^–29^
Breast cancer GWAS	469	157	0.0144
Pancreatic cancer GWAS	468	170	0.0120
Prostate cancer GWAS	469	172	0.0053
Metformin GWAS^c^	458	177	0.0181

^a^ For the five GWAS data sets, each number denotes the number of genes with genotyping data in corresponding GWAS data. The T2D genes were extracted from the GWAS Catalog database and the cancer genes were obtained from Cancer Gene Census.

^b^ For disease genes, the hypergeometric test was performed by comparing with all protein-coding genes in the human. For GWAS data, the hypergeometric test was performed by comparing with genotyping data in the corresponding GWAS data set.

^c^ Metformin GWAS: the GWAS for identifying genetic variants associated with survival among cancer patients with T2D using metformin.

### Major steps to improve DSPathNet’s performance

In order to generate a complete and reliable SPNetwork, we extensively collected the metformin related genes, rigorously selected the expressed genes induced by metformin, and comprehensively compared the performance using T2D GWAS data after the SPNetwork generation. For each step, we provide the detailed information as below.

#### Collection of metformin upstream genes

We first collected the 46 genes related to metformin from two databases DrugBank and PharmGKB. Among them, 21 genes existed in the 4,367 genes in the human SPNetwork. To collect the metformin-related genes to the maximum extent possible, we further performed literature mining on the MEDLINE abstracts to identify the gene entities that have a relation with metformin by calculating the semantic distance among the hidden topics uncovered by Latent Dirichlet Allocation (LDA) model [37]. We obtained 29 genes. Among them, ten overlapped with the 46 genes and 19 were uniquely identified by the literature searching method. Of these 19 genes, 15 were found in the human SPNetwork (S2 Table, S1 Fig). Collectively, we obtained a total of 65 genes that were regarded as metformin upstream genes, among which 36 genes could be mapped to human SPNetwork.

#### Inference of metformin downstream genes

**Fig 2:**
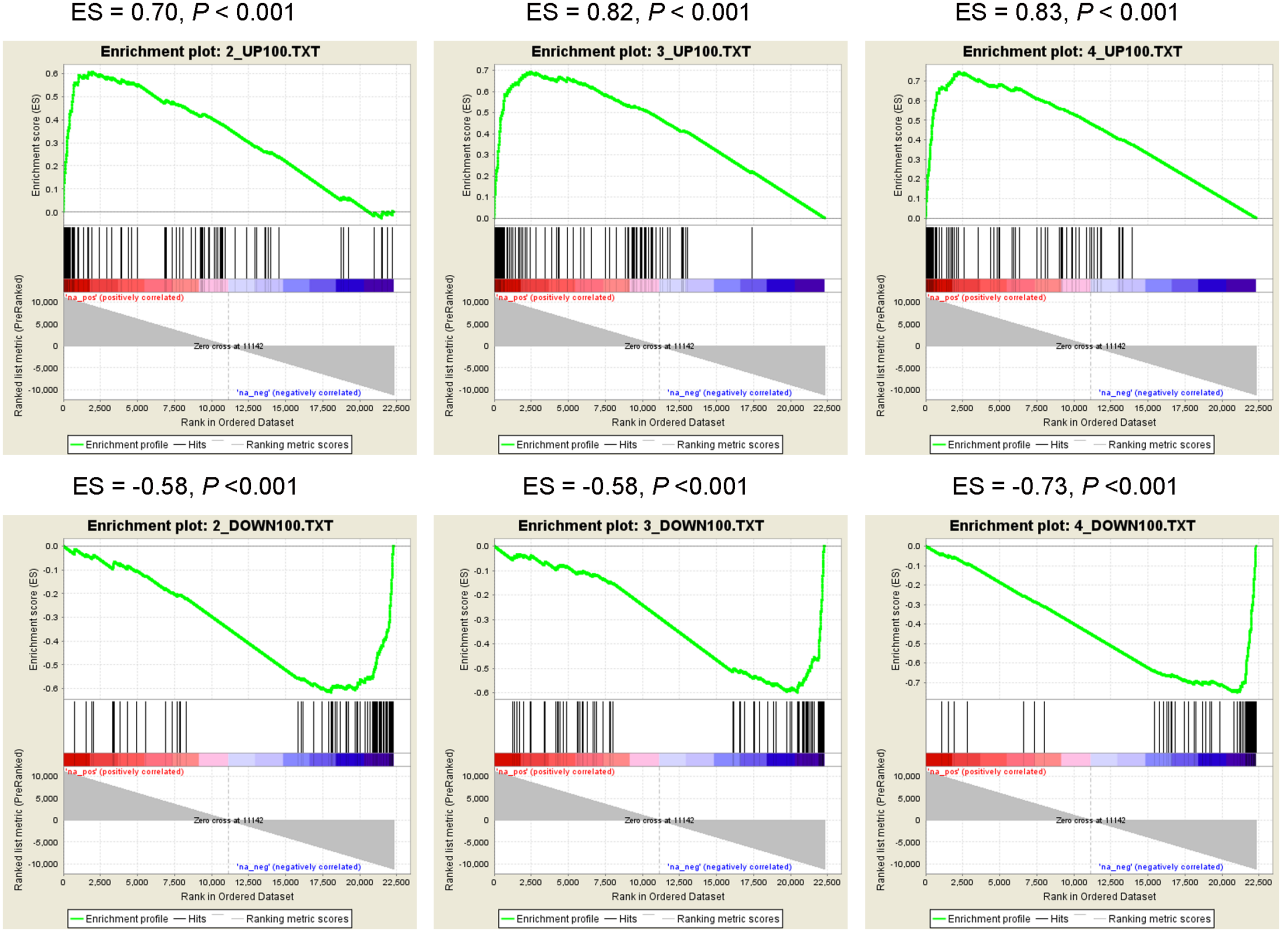
Gene Set Enrichment Analysis (GSEA) enrichment score curves of metformin-induced probes in three treatments vs. treatment 1. The four sets of probes of metformin treatments were obtained from the gene expression profiles from Connectivity Map. The three treatment instance IDs are 2, 3, and 4. The graphs on the top panels represent the ranked, non-redundant, and up-regulated probes in the second, third, and fourth treatment groups compared with probes in the first treatment group. The graphs on the bottom panels represent the ranked, non-redundant, and down-regulated probes in second, third, and fourth treatment groups compared with probes in the first treatment group. In each graph, probes on the far left (red) correlated with the most up-regulated probes in the treatment 1 and probes on the far right (blue) correlated with the most down-regulated probes in treatment 1. In each graph, the vertical black lines indicate the position of each of the probes of the studied probe set in the ordered, non-redundant data set. The green curve denotes the ES (enrichment score) curve, the running sum of the weighted enrichment score in GSEA.

We inferred the metformin downstream genes based on gene expression data in cancer cells after metformin treatment from Connectivity Map (CMap) (build 02) [15]. Among the ten gene expression datasets of metformin treatments (S3 Table), four had significantly consistent with each other (absolute value of the enrichment score > 0.5 and FDR *q*-value < 0.001) (Fig 2 and S2 Fig) by performing the gene set enrichment analysis (GSEA) [38]. Then, based on the top and bottom 100 probes for the four treatments, we identified 140 up-regulated and 215 down-regulated genes, respectively. From these genes, we identified 29 TFs whose targets were significantly enriched in up-regulated genes and 38 TFs whose targets were significantly enriched in down-regulated genes (Hypergeometric test *P*-value < 0.05) compared to the pairs of TFs and their targets (Materials and Methods). There was one TF (TEAD4) shared between the two sets of TFs. Thus, we identified 66 TFs in total (S4 Table). Among these TFs, only one TF (JUN) was observed in the list of the up-regulated genes and two TFs (SMAD3 and NR1I2) in the down-regulated genes. Our observation is in general agreement with previous reports that many TFs are not regulated at the transcriptional level [39,40].

#### Generation and evaluation of metformin-specific SPNetwork

**Fig 3:**
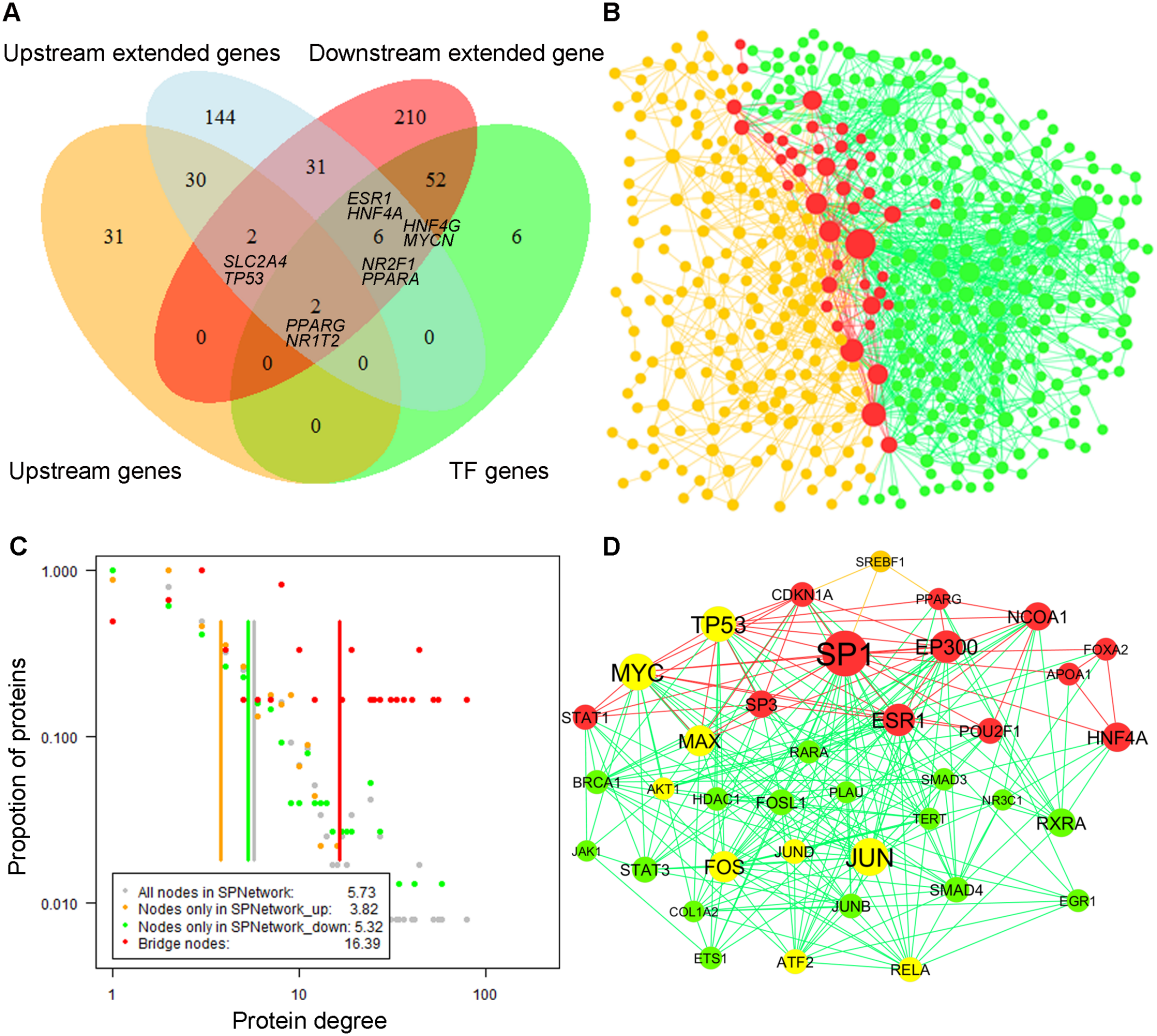
Metformin-specific signaling pathway network (SPNetwork). A) A four-way Venn diagram summarizes the number of shared genes among metformin upstream genes represented by ‘Upstream genes’, metformin downstream genes (‘TF genes’), metformin upstream extended genes in the metformin upstream network (‘Upstream extended genes’), and metformin downstream extended genes in the metformin downstream network (‘Downstream extended genes’). B) Metformin-specific SPNetwork with 477 nodes and 1366 edges. The nodes and edges in orange correspond to nodes and edges only in the metformin upstream network. The nodes and edges in green correspond to the nodes and edges only in the metformin downstream network. And the nodes and edges in red correspond to the nodes and edges common to the metformin upstream network and the metformin downstream network. C) Degree distributions and average degrees (vertical lines) of the four gene sets in the metformin-specific SPNetwork. The four gene sets are 41 common nodes, 174 nodes only in the metformin upstream network (SPNetwork_up), 262 nodes only in the metformin downstream network, all 477 nodes in the metformin-specific SPNetwork (SPNetwork_down). The Y-axis represents the proportion of proteins having a specific degree. D) The subnetwork of the 38 hub nodes extracted from metformin-specific SPNetwork. The legends for orange nodes and edges, red nodes and edges, and green nodes and edges are same as those in the subFig B. The nodes in yellow correspond to the genes that exist in the pathway ‘MAPK signaling pathway’ according to KEGG annotation.

We noticed that only two genes (*PPARG* and *NR1I2)* were common between the metformin upstream gene list and the metformin downstream gene list (Fig 3A). The observation indicated that some of the key components in the metformin signal transduction cascade were missed in the two sets of metformin-related genes. To address this issue, we employed a two-step strategy of random walk-based propagation to recruit more genes via a sequential two-step strategy from the human SPNetwork (Materials and Methods). Table 2 summarizes the number of nodes and edges generated at each step. Through two network movements, we obtained 215 upstream extended genes and 303 downstream extended genes. Then we generated one upstream network by the direct links of metformin upstream extended genes (SPNetwork_up) and one downstream network by the direct links of metformin downstream extended genes (SPNetwork_down). They had 41 common nodes and 84 common links. After merging the two networks by their common nodes and common links, we obtained a metformin-specific network with 477 nodes and 1,366 edges.

**Table 2:** Summary of genes and hypergeometric tests at each step in the process of metformin-specific SPNetwork construction.

	#. genes			Network			*P*-value^b^
	Upstream	Downstream	Overlap	#. nodes ^a^ (All/largest)	#. nodes with *P* <0.05	#. edges	
Starting seed	65	66	2	98/74	36	179	0.02
Longitudinal movement	103	125	9	219/151	74	384	0.01
Lateral movement	215	303	41	477/473	169	1,366	3.08 ×10^-5^

^a^ The first number denotes the number of the nodes in the whole network while the second number denotes the number of nodes of the largest subnetwork in the network.

^b^The *P*-value was calculated from the hypergeometric test by comparing the number of T2D-related genes that have at least one SNP with *P*-value less than 0.05d with all genotyping genes in the T2D GWAS data based on.

Compared to the two common genes between the metformin upstream genes and downstream genes, the overlap was increased 20.5 times (Fig 3A). Among the 41 nodes, besides the two common genes (*PPARG* and *NR1T2*), two genes belonged to the metformin upstream genes (*SLC2A4* and *TP53*), and six genes belonged to metformin downstream genes (*ESR1*, *HNF4A*, *HNF4G*, *MYCN*, *NR2F1*, and *PPARA*). The remaining 31 genes (75.6%) were novel linkers, suggesting they might play important roles in metformin action. Considering that these 41 nodes act as bridges to link the SPNetwork_up and SPNetwork_down, we defined them as bridge nodes.

To assess the ability of recruiting disease genes of each step, we produced one corresponding network for each step and perform disease gene enrichment analysis based on the T2D GWAS data from the Wellcome Trust Case Control Consortium (WTCCC) T2D study [31]. The concept is based on that the more significant enrichment of disease genes the corresponding network has, the more powerful the network-generating method is. Table 2 summarizes the corresponding evaluation *P*-values. Starting from the unique 129 genes of metformin upstream genes and TF genes, a network with 98 nodes and 179 edges was produced (S3 Fig). The largest module contained 74 nodes and 178 edges, which indicated that metformin upstream genes and downstream genes could regulate each other to a certain degree. Among 98 nodes, 93 had genotyping data in T2D GWAS, of which 36 genes belonging to the T2D-related genes. Compared with all genes with genotyping data in the GWAS, the hypergeometric test *P*-value was 0.02.

In the first step (longitudinal movement), from upstream genes, we obtained 103 genes, of which 36 were upstream genes and 67 were novel genes (S4A Fig). From metformin downstream genes, we obtained 125 genes that contained 62 metformin downstream genes and 63 novel genes (S4B Fig). Between the 103 genes and the 125 genes, there were nine common genes. Then, a subnetwork was created by their direct interactions with 219 nodes (S5 Fig). In the network, the largest module included 151 (68.9%) of the 219 genes, indicating that about one-third of genes (68, 31.1%) could not be recruited in the biggest subnetwork. In addition, among the 219 nodes, 207 had genotyping data, in which 74 were T2D-related genes. Compared with all genes with genotyping data in the T2D GWAS, this network module is statistically enriched with genes having small *P* values (Hypergeometric test, *P*-value: 0.01), but the significance is not very strong.

In the second step (lateral movement), from the metformin upstream longitudinal genes (103), we obtained 215 genes, including 34 metformin upstream genes, 60 metformin upstream longitudinal genes, and 121 novel genes (S4A Fig). From the metformin downstream longitudinal genes (125), we obtained 303 genes comprised of 60 metformin upstream genes, 56 metformin upstream longitudinal genes, and 187 novel genes (S4B Fig). After merging their direct interactions, we obtained a network with 477 nodes and 1,366 edges. Among the 477 nodes, 473 nodes (99.2%) formed a big module. The network had the strongest association with T2D-related genes (*P*-value: 3.08 ×10^–5^). More importantly, the number of common nodes increased to 41. Therefore, the crossing movement strategy is promising to capture the cascade of signal flow and complexity of cross-talking among different pathways involved in signal transduction from the upstream genes and downstream genes.

### Metformin-specific SPNetwork provides a valuable source for understanding metformin action

The final metformin-specific SPNetwork generated above comprised 477 nodes and 1,366 edges (Fig 3B, S5 Table). Among the 477 nodes, 215 belonged to metformin upstream network, while 303 belonged to metformin downstream network. There were 41 bridge nodes between them. Thus, 174 genes were unique to the metformin upstream network, and 262 genes were unique to the metformin downstream network. From here, we refer to the three gene sets as upstream genes (number of genes: 174), downstream genes (262), and bridge genes (41) for follow-up network topological and functional analyses.

To explore the topological properties of this SPNetwork, we calculated node degrees (connectivity) and their distribution [41]. In this network, degree values of nodes ranged from 1 to 79 and the average degree was 5.73. The degree distribution was strongly right-skewed, indicating that most nodes had a low degree and only a small portion of the nodes had a high degree (Fig 3C). The nodes with a high degree act as hubs in the network and hold the whole network together [41]. In biological networks, hubs are more likely to be essential genes [42] and disease genes [43–45]. Using the hub defining method proposed by Yu et al. [46], we determined 38 hubs whose degrees were larger than 14. Among them, one gene (*PPARG*) belonged to both metformin upstream and downstream gene sets, two genes (*TP53* and *SREBF1*) were metformin upstream genes, 13 belonged to the metformin downstream gene set only, and 22 were novel genes. After extracting these hubs from metformin-specific SPNetwork, we generated a hub-centered subnetwork (Fig 3D). Among the 38 hubs, 19 (50.00%) are included in ‘pathway in cancer’ and 9 (23.68%) in ‘MAPK signaling pathway’ according to KEGG pathway annotation. The MAPK signaling pathway plays important roles in the pathology of both cancer [47] and diabetes [48]. Thus, the 477 genes had two genes belonging to metformin upstream and downstream genes, 33 to the metformin upstream genes, 58 to the metformin downstream genes, and 384 novel genes (S6 Table). The novel genes may provide a valuable resource for further investigation of the pathology of T2D and cancer, and the metformin action.

We further examined pathway enrichment in these 477 nodes based on KEGG pathway annotation using the online tool WebGestalt [49]. We identified 69 significant pathways (adjusted *P-*value < 1.00 × 10^–4^) (S7 Table). According to the KEGG pathway first-level category annotation (Materials and Methods), 12 pathways belonged to ‘environmental information processes,’ nine to ‘cellular processes,’ 18 to ‘organismal systems,’ and 29 to ‘human disease.’ Among these 12 environmental information processes pathways, eight were signal transduction pathways, of which the top three pathways were ‘MAPK signaling pathway’ (32genes, adjusted *P*-value: 3.39 × 10^–22^), ‘mTOR signaling pathway’ (13 genes, adjusted *P*-value: 6.39 × 10^–14^) and ‘ErbB signaling pathway’ (15 genes, adjusted *P*-value: 1.89 × 10^–13^). Among the 18 pathways related to organismal systems, five belonged to the endocrine system, of which the top three pathways were ‘adipocytokine signaling pathway’ (22 genes, adjusted *P*-value: 3.19 × 10^–25^), ‘PPAR signaling pathway’ (22 genes, adjusted *P*-value: 5.36 × 10^–25^), and ‘insulin signaling pathway’ (23 genes, adjusted *P*-value: 1.91 × 10^–19^). Among the 29 pathways related to human disease, 15 were directly related to cancer. Importantly, the pathway ‘type II diabetes’ (10 genes, adjusted *P*-value: 1.12 × 10^–10^) and the ‘maturity onset diabetes of the young’ (8 genes, adjusted *P*-value: 1.94 × 10^–10^) were among the enriched pathways. Together, the evidence indicates that the metformin-specific SPNetwork involves both diabetes and cancer at the pathway level.

### Network topological and functional properties of bridge genes

In the metformin-specific SPNetwork, there were 41 genes (bridge genes) common to both the metformin upstream and downstream networks. As mentioned above, most of them (31, 75.6%) were novel linkers (S6 Table). To interrogate their roles, we compared them with upstream genes (174) and downstream genes (262) via network topological and functional analyses, as described below.

#### Bridge genes tended to have higher degree

In the metformin-specific SPNetwork, the average degree of these bridge genes was 16.39, significantly higher than that of upstream genes (3.82, Wilcoxon's test, *P*-value: 2.41×10^–6^) or that of downstream genes (5.32, *P*-value: 1.69 × 10^–6^) (Fig 3C). The result indicated that the bridge genes strongly connected in the metformin-specific SPNetwork. In line with this, the bridge nodes are more likely to be the hubs: 15 out of 41 (36.9%) as compared to 1 out of 174 upstream genes (0.57%) or 22 in 262 downstream genes (7.6%). These 15 genes were *SP1* (degree: 79 in the metformin-specific network), *MYC* (55), *TP53* (52), *EP300* (44), *ESR1* (44), *MAX* (40), *HNF4A* (36), *NCOA1* (33), *SP3* (31), *POU2F1 (27)*, *STAT1* (25), *CDKN1A* (24), *APOA1* (19), *FOXA2* (19), and *PPARG* (17). This observation indicates that these nodes might play important roles to maintain the network topology that is important for biological function.

#### Bridge genes had different functional tendencies

**Fig 4:**
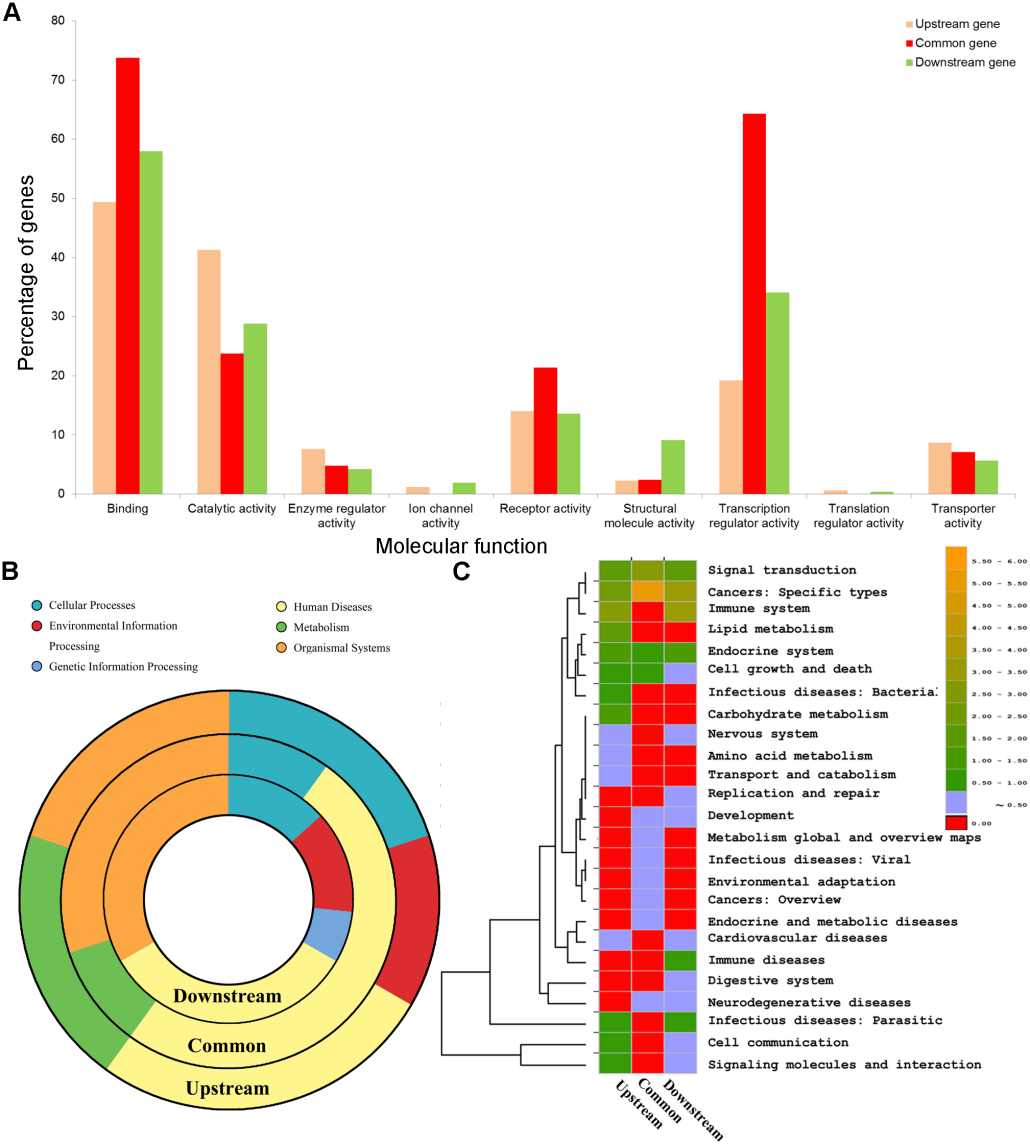
Functional comparison of the common genes, upstream network genes, and downstream network genes. The common genes were those found in both metformin upstream network and downstream network. The upstream network genes were those only belonging to the metformin upstream network. The downstream network genes were those only belonging to the metformin downstream network. A) Proportion of genes of interest in Gene Ontology (GO) molecular function domain. B) Comparison of proportion of enriched pathway in the three gene sets at the first-level category of KEGG annotation. C) The clustering of enriched pathways for the three gene sets at second-level category of KEGG annotation.

To further explore the functional characteristics of these bridge genes, we first compared them with upstream genes and downstream genes based on the GO Molecular Function domain using the online tool PANTHER Classification System [50] (Fig 4A). The proportion of genes in the following three GO terms were higher in the bridge genes than that in the upstream or downstream genes: binding (GO:0005488), receptor activity (GO:0004872), and transcription regulator activity (GO:0030528). However, for the following three GO terms, the proportion of upstream genes was significantly higher than that in other two gene sets: catalytic activity (GO:0003824), enzyme regulator activity (GO:0030234), and transporter activity (GO:0005215). For the downstream genes, only one GO term, structural molecule activity (GO:0005198), had a higher proportion.

We also examined the enriched pathways in the three sets of genes according to the KEGG enrichment analyses using the tool WebGestalt. By applying an adjusted *P*-value of less than 0.05, we found that 92 pathways were significantly enriched in the 174 upstream genes, 105 pathways in the 262 downstream genes, and 28 pathways in 41 common genes (S8 Table). To simplify the comparison, we categorized them into seven categories at the first level and 43 categories at the second level in the KEGG pathway annotation system (Materials and Methods). To represent the relative abundance of the pathways, we further calculated a Z-score for each category at the second level (Materials and Methods). Accordingly, among the 92 pathways for upstream genes, 73 pathways were grouped into the five first-level categories and 15 second-level categories (Z-score > 0). Among 106 enriched pathways in downstream genes, 86 pathways were grouped in five first-level categories and 15 second-level categories (Z-score > 0). All of the 28 enriched pathways in 41 common genes were categorized into five first-level categories and 11 second-level categories (Z-score > 0) (S9 Table). Fig 4B summarizes the comparison of the three sets of genes at the first-level category. We observed that each of the three sets of genes had their own participating tendency in particular biological processes. For example, among the 73 enriched pathways in the upstream genes, 15 (20.55%) belonged to the metabolism category, which was substantially higher than that in the common genes (1, 3.57%) or that in the downstream genes (0). Among the 28 enriched pathways in the common genes, 15 (53.57%) belonged to the human diseases, which was higher than that in the upstream genes (19, 26.03%) or that in the downstream genes (32, 37.21%). Fig 4C further shows the pathway comparison of the three sets of genes at the second level. While all genes in the three gene sets were enriched in the pathways related to cancer, compared to upstream and downstream genes, the bridge genes were the likeliest to be involved in the cancer-related pathways.

Among the 41 bridge nodes, 25 were TFs according to TRANSFAC database. Among them, eight (ESR1, HNF4A, HNF4G, MYCN, NR1I2, NR2F1, PPARA, PPARG) were inferred metformin-related TFs based on metformin-induced gene expression data and the remaining 16 TFs were identified as the novel linkers between metformin upstream and downstream network. They were ARNTL, CDX2, EP300, FOXA2, MAX, MYC, NCOA1, PHOX2A, POU2F1, RORA, RXRG, SP1, SP3, STAT1, TGIF1, and USF2. Among them, MYC is encoded by a well-known oncogene that acts as a pluripotent modulator of transcription during normal cell growth and proliferation [51]. Interestingly, several other TFs cooperate with MYC under some particular conditions such as CDX2 [52], MAX [53], SP1 [54–57], SP3 [58], and STAT1 [54]. For example, CDX2, one caudal-related homeobox transcription factor, mediates E-selectin ligand expression in colon cancer cells with MYC together [52].

In summary, our network and functional analyses indicated that these common genes act as bridges between the metformin upstream and downstream networks so that they might act in metformin-specific SPNetwork. Therefore, these bridges genes, especially the novel genes, are warranted for further investigation of their roles in the signal transduction cascade of metformin action.

### Metformin-specific SPNetwork is significantly enriched with T2D associated genes

Since metformin is a well-studied drug for T2D treatment, the metformin-specific SPNetwork was expected to contain genes that have genetic association with T2D. To examine this expectation, we comprehensively performed enrichment analysis using two sets of genes. The first one contained 131 genes collected from 66 T2D GWAS studies curated by the NHGRI GWAS Catalog database (April 1, 2014) [29]. Those genes have been reported to be significantly associated with T2D based on GWA studies. Here, we selected these genes having at least one SNP with *P*-value less than 1.0 × 10^–8^ as T2D associated genes. The second set included the T2D-related genes from the WTCCC T2D study [31] as mentioned above.

Among the 477 nodes in the metformin-specific SPNetwork, 11 genes were found in the first set of 131 genes. Compared to the human protein-coding genes (20,716), the network was significantly enriched for T2D associated genes (Hypergeometric test, *P*-value: 1.36 × 10^–4^). Similarly, among the 131 T2D disease genes, 43 existed in the human SPNetwork (4,367). Thus, compared to all nodes in the human SPNetwork, the metformin-specific SPNetwork was significantly enriched for T2D associated genes too (*P*-value: 3.62 ×10^–3^). These 11 genes were *CDKN2B*, *HNF1A*, *HNF4A*, *IRS1*, *ITGB6*, *KCNJ11*, *LEP*, *PPARD*, *PPARG*, *SND1*, and *TCF7L2*. Among them, *KCNJ11*, *PPARG*, and *TCF7L2* have the strongest genetic association among genes that appear in the T2D GWAS studies based on a comprehensive review [59].

**Fig 5:**
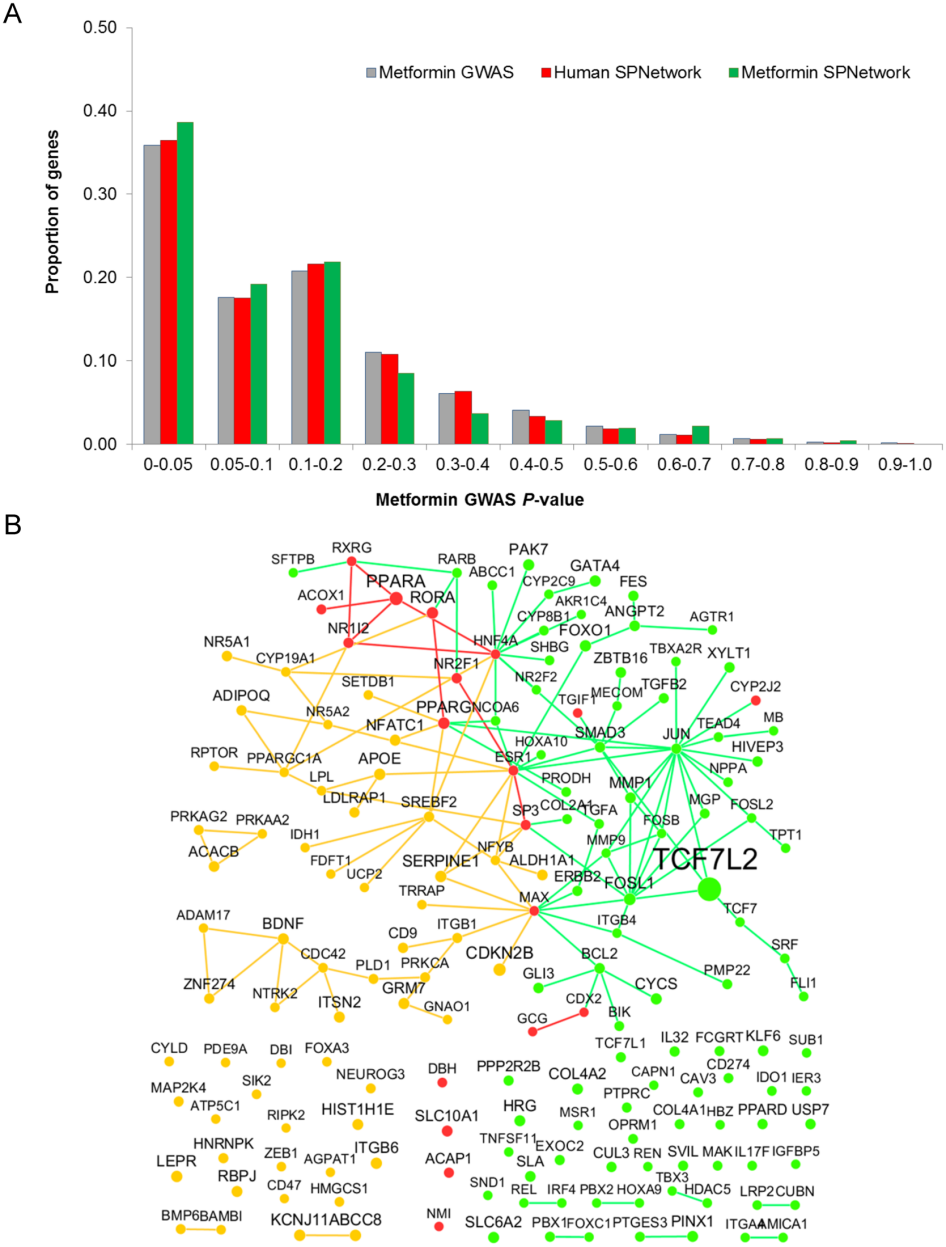
(A) Comparison of gene-level *P*-value distribution in T2D GWAS among three gene sets from the metformin-specific SPNetwork, human SPNetwork, and genes covered by T2D GWAS. (B) Interactions were extracted from metformin-specific SPNetwork. These interactions occur between T2D-related genes, namely, their smallest *P*-value less than 0.05 in T2D GWAS. The legends for orange nodes, red nodes, and green nodes are same as in Fig 3.

Among the 477 genes in metformin-specific SPNetwork, 445 had genotyping data from WTCCC T2D GWAS dataset. Among them, 169 genes belonged to T2D-related genes. Compared with all genes with genotyping data in the GWAS, the network was significantly enriched with T2D-related genes (Hypergeometric test *P*-value: 3.08 ×10^–5^). We further compared the 169 genes with the genes having genotyping data in the human SPNetwork. Among the 4,367 nodes in the human SPNetwork, 3,446 genes had genotyping data, in which 1,048 genes were T2D-related genes. Thus, the metformin-specific SPNetwork was significantly enriched for the T2D-related genes as compared to the whole human SPNetwork in this study (*P*-value: 7.47 ×10^–3^). Fig 5A shows the comparison of *P*-value distributions of genes in whole GWAS data (T2D GWAS), human SPNetwork, and metformin-specific SPNetwork. These comparisons indicate that the network is enriched with genes that might be involved in the pathology of T2D.

We further generated a subnetwork for 169 nominally significant genes with T2D (Fig 5B) by their direct links. Among the 169 genes, 50 genes had SNPs whose *P*-values were less than 0.01 in the WTCCC T2D GWAS. In addition, there were six genes observed in both the 131 GWAS Catalog genes and the 169 genes; they are *CDKN2B*, *ITGB6*, *KCNJ11*, *PPARD*, *PPARG*, and *TCF7L2*. Among them, the SNP rs4506565 in gene *TCF7L2* has the strongest significance (*P* = 5.68 ×10^–13^). *TCF7L2* encodes a transcription factor that regulates the transcription of several genes. It is a key element in the WNT signaling pathway, which has been reported to contribute to T2D risk significantly [59].

### Metformin-specific SPNetwork is enriched with cancer genes

Above pathway analysis indicated that the metformin-specific SPNetwork was significantly associated with cancer-related pathways. Here, we further examined if the SPNetwork is enriched with cancer genes from four data sets. The first one included 509 cancer genes downloaded from the Cancer Gene Census (December 11, 2013, http://cancer.sanger.ac.uk/cosmic/census). Among them, 64 genes were included in the metformin-specific SPNetwork. Compared to all human genes or the protein-coding genes in the human SPNetwork, the network was significantly enriched with cancer genes (Hypergeometric test, *P*-value: 1.64 × 10^–29^ and 6.48 × 10^–8^, respectively). Interestingly, 3 of the 64 genes (*HNF1A*, *PPARG*, and *TCF7L2*) were in the T2D GWAS Catalog gene list, and 21 genes belonged to 169 T2D-related genes (see above). This observation strongly indicates that metformin may affect the shared genetic risk factors between T2D and cancer. Such information provides clues for how metformin acts in T2D and cancer treatments. This observation also provides evidence for epidemiological studies of metformin in both T2D and cancer [50].

Additionally, we performed the GSEA of the metformin-specific SPNetwork using three cancer GWAS datasets from the Cancer Genetic Markers of Susceptibility (CGEMS) projects (breast cancer [32], pancreatic cancer [33], and prostate cancer [32]). Table 1 summarizes the corresponding gene numbers in each GWAS dataset. Compared with all genes with genotyping in each GWAS dataset, the metformin SPNetwork was slightly significantly enriched in nominally significantly associated genes (Hypergeometric test *P*-values: 0.0144, 0.0120, and 0.0053 for breast, pancreatic, and prostate cancer, respectively). Though the results of these statistical tests are not as robust as that of the genotyping data from the T2D GWAS study, the results confirm that the metformin-specific SPNetwork was enriched with genetic factors associated with cancer development.

### Metformin-specific SPNetwork is enriched with genes associated with overall survival of cancer patients with T2D using metformin

From above analyses, the metformin-specific SPNetwork is enriched with genes associated with T2D and cancer. Several studies over the last few years have demonstrated that patients using metformin have reduced cancer risk and improved cancer survival in T2D patients [24,26,60,61]. Thus, we evaluated whether metformin-specific network enrich genes associated with cancer survival among cancer patients with T2D using metformin. In this study, we took advantage of GWAS data of cancer subjects with T2D treated with metformin from BioVU [34,62] (Materials and Methods). Hereafter, this dataset is referred as “metformin GWAS.” Among the 477 nodes in the metformin-specific SPNetwork, 458 genes had genotyping and 177 genes were nominally significantly (*P*-value < 0.05) associated with T2D with better survival. Compared with all genes with genotyping data in the metformin GWAS data, the metformin-specific SPNetwork was enriched with nominally significant genes too (Hypergeometric test, *P*-value: 0.0181). We further compared the *P*-value distribution of metformin GWAS data for three gene sets: the metformin-specific SPNetwork, human SPNetwork, and all genes in metformin GWAS data set (S6 Fig). The genes in the metformin SPNetwork had the highest proportion of *P*-values (*P*-value < 0.05) in metformin GWAS data at the gene level.

Among the 177 genes, 81 genes were included in the 169 genes whose smallest *P*-values were less than 0.05 in T2D GWAS data. While most of them did not link to each other (S7 Fig), these 81 genes directly linked to other 175 genes to form a subnetwork that included 256 nodes and 910 edges. This feature indicated that the 81 genes and their direct interactors dominated the metformin-specific SPNetwork. For example, the 256 nodes accounted for 53.7% of all nodes and the 910 edges accounted for 66.6% of all edges in the metformin-specific SPNetwork. Additionally, among the 81 genes, 17 belonged to ‘pathway in cancer’: *COL4A1*, *COL4A2*, *ERBB2*, *GLI3*, *ITGB1*, *MECOM*, *MMP1*, *PLD1*, *PRKCA*, *RARB*, *RXRG*, *SMAD3*, *TCF7L1*, *TCF7L2*, *TGFA*, *TGFB2*, and *ZBTB16*. Collectively, the above observations indicate that the network was enriched in genes that might contribute to overall survival among cancer patients with metformin therapy.

### Crosstalk subnetwork intertwines the key genes for metformin action in T2D and cancer

**Fig 6:**
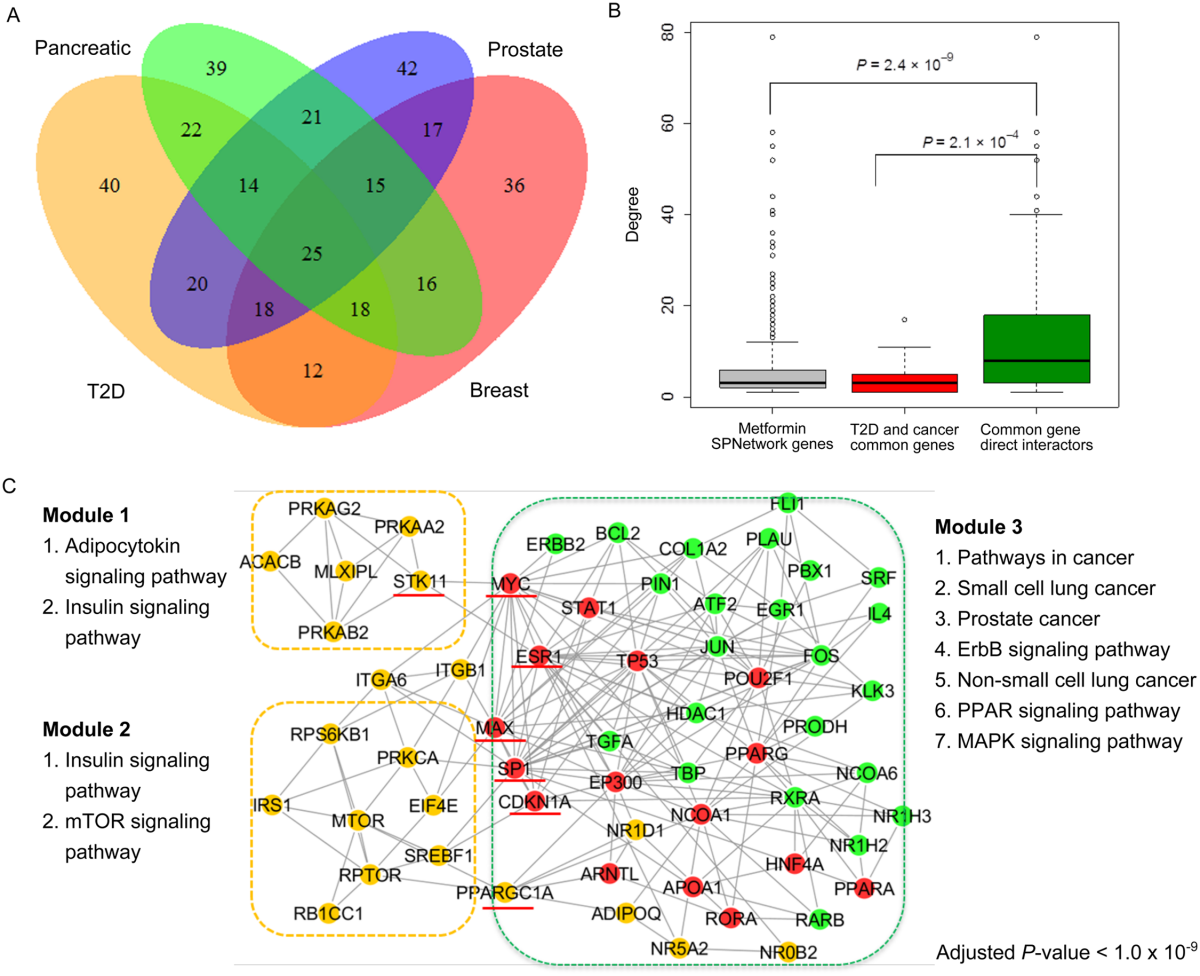
Common genes and a crosstalk subnetwork between T2D and cancer. (A) The four-way Venn diagram summarizes the number of shared genes among the four gene sets with smallest *P*-value less than 0.05 in the T2D GWAS and the three types of cancer GWAS data sets (breast, pancreatic, prostate) in metformin-specific SPNetwork. (B) Degree comparison of common genes among the four gene sets in A, common genes’ direct interactors, and all genes in metformin-specific SPNetwork. (C) A crosstalk subnetwork of metformin action for T2D and cancer with three modules and enriched pathways. The legends for orange nodes, red nodes, and green nodes are same as in Fig 3. The nodes with underlines are key components in the metformin signal transduction process.

From above analyses, we observed that the metformin-specific SPNetwork was enriched with genes associated with T2D and cancer, and genes associated with metformin-associated cancer survival. To gain more insights into how metformin act in T2D and cancer treatment, we generated a subnetwork to synopsis the crosstalk between T2D and cancer based on the common genes with nominal significance (*P*-value < 0.05) among the four GWAS data sets (T2D, CGEMS breast cancer, pancreatic cancer, and prostate cancer). There were 25 genes common to all the four gene sets (Fig 6A), and there were only five edges in the metformin-specific SPNetwork (S8 Fig). By further examining degree distributions of the common 25 genes and their direct interactors (71 genes), we found that their interactors had significantly more interactions than the 25 genes as well as all the genes in the metformin-specific SPNetwork (Wilcoxon’s test *P*-value: 2.1 × 10^–4^ and 2.4 × 10^–9^, respectively) (Fig 6B). The 25 genes included one hub (PPARG) while the 71 genes included 21 of the 38 hub nodes in the metformin-specific SPNetwork. Similarly, the 25 genes contained three bridge nodes while the 71 genes contained 15 of the 41 bridge nodes between metformin upstream and downstream network. These observations indicate that the interactors of the 25 common nodes were more likely to play important roles for signal transduction.

Starting with the 25 genes and their 71 interactors, we assembled a subnetwork by their direct links among 96 nodes. The subnetwork comprised 96 nodes and 269 edges (S9 Fig). To further explore the metformin treatment mechanisms in T2D and cancer through the protein modules, we utilized software CFinder to perform network cluster and community analysis [63]. We required each node in one module participate at least one 3-vertex clique. Accordingly, we obtained three modules, which contained 6, 9, and 51 genes, respectively (S10 Fig). We found no gene shared between the first and second modules, but one gene (*STK11*) common to the first and third modules, or five genes (*EIF4E*, *PPARGC1A*, *PRKCA*, *RPS6KB1*, and *SREBF1*) common to the second and third modules. All the genes of the first and second modules belonged to metformin upstream network while most of the genes in the third module belonged to metformin downstream network. We merged them to form a network, which included 60 nodes and 210 edges (Fig 6C). Since this subnetwork was generated from common genes to T2D and cancer genotyping data, we defined it as a crosstalk subnetwork of metformin action in T2D and cancer.

We realized that, if we removed the nodes (CDKN1A, ESR1, MAX, MYC, PPARGC1A, STK11, and SP1), the connections among three modules would be lost (S11 Fig). Among them, three (MAX, MYC, and SP1) were both the bridge nodes and hub nodes. Therefore, these seven nodes might be functionally critical in the metformin signal transduction cascade. To further explore how the three modules and the seven key nodes might be related to metformin treatment in term of biological function meaning, we performed the KEGG pathway enrichment analysis on each module. Table 3 summarizes the enriched pathways for each module (adjusted *P*-value < 1.0 × 10^–4^). We labeled the enriched KEGG pathways (adjusted *P*-value < 1.0 × 10^–9^) for each module in Fig 6C.

**Table 3:** KEGG pathways overrepresented in genes in three modules of the reduced common network between T2D and cancer for metformin.

Pathway	#. interest genes	Adjusted *P*-value^a^
***Module # 1***		
Adipocytokine signaling pathway	5	2.01 × 10^-13^
Insulin signaling pathway	4	3.00 × 10^-9^
Hypertrophic cardiomyopathy (HCM)	3	1.83 × 10^-7^
***Module # 2***		
Insulin signaling pathway	7	1.05 × 10^-15^
mTOR signaling pathway	4	1.18 × 10^-9^
Adipocytokine signaling pathway	3	1.04 × 10^-6^
ErbB signaling pathway	3	1.65 × 10^-6^
Aldosterone-regulated sodium reabsorption	2	6.64 × 10^-5^
Type II diabetes mellitus	2	7.25 × 10^-5^
Acute myeloid leukemia	2	8.77 × 10^-5^
***Module # 3***		
Pathways in cancer	19	6.89 × 10^-26^
Small cell lung cancer	8	2.94 × 10^-12^
Prostate cancer	7	2.06 × 10^-10^
ErbB signaling pathway	7	2.06 × 10^-10^
Non-small cell lung cancer	6	6.09 × 10^-10^
PPAR signaling pathway	6	2.54 × 10^-9^
MAPK signaling pathway	8	8.80 × 10^-9^
Huntington's disease	7	1.66 × 10^-8^
Focal adhesion	7	2.74 × 10^-8^
Colorectal cancer	5	7.08 × 10^-8^
Hepatitis C	6	7.15 × 10^-8^
Adipocytokine signaling pathway	5	9.45 × 10^-8^
Leishmaniasis	5	1.17 × 10^-7^
Thyroid cancer	4	1.76 × 10^-7^
Bladder cancer	4	7.65 × 10^-7^
Cell cycle	5	1.46 × 10^-6^
Wnt signaling pathway	5	3.53 × 10^-6^
Glioma	4	3.76 × 10^-6^
Pancreatic cancer	4	4.82 × 10^-6^
Chronic myeloid leukemia	4	5.43 × 10^-6^
Circadian rhythm—mammal	3	6.86 × 10^-6^
TGF-beta signaling pathway	4	8.65 × 10^-6^
Osteoclast differentiation	4	4.40 × 10^-6^
Toxoplasmosis	4	4.75 × 10^-5^
Insulin signaling pathway	4	5.44 × 10^-5^
mTOR signaling pathway	3	7.46 × 10^-5^
Endometrial cancer	3	7.46 × 10^-5^
Jak-STAT signaling pathway	4	7.67 × 10^-5^

^a^Adjusted *P*-value was calculated from hypergeometric test following by Benjamini-Hochberg multiple testing correction.

In the first and second modules, there were two common pathways: adipocytokine signaling pathway and insulin signaling pathway. Adipocytokine signaling pathway was the top pathway in the first module (adjusted *P*-value: 2.01 × 10^–13^). The adipocytokine is a group of cytokines secreted by adipose tissue, which contributes to the development of insulin resistance, T2D, and cardiovascular disease [64,65]. The insulin signaling pathway, the top pathway in the second module, plays important roles in many complex diseases such as diabetes, obesity [66], and neurological disorders [67]. In addition, the mTOR signaling pathway and ErbB signaling pathway were also enriched in the second module. There were 28 pathways enriched in the third community. According to KEGG pathway annotation at the second level, 15 of these 28 pathways belonged to human disease, six to signal transduction, and three belonged to the endocrine system, one to cell communication, one to cell growth and death, one to development, and one to environmental adaptation. Among the 15 human disease related pathways, 11 were for specific types of cancer. Therefore, the three modules reflected different biological processes involved in T2D and cancer. Additionally, the pathway analyses highlighted the seven nodes that are not only topological linkers but also functional linkers in the crosstalk SPNetwork of metformin action in T2D and cancer.

### Literature mining further reveals a novel MYC-centered pathway may play critical roles in metformin action

**Fig 7:**
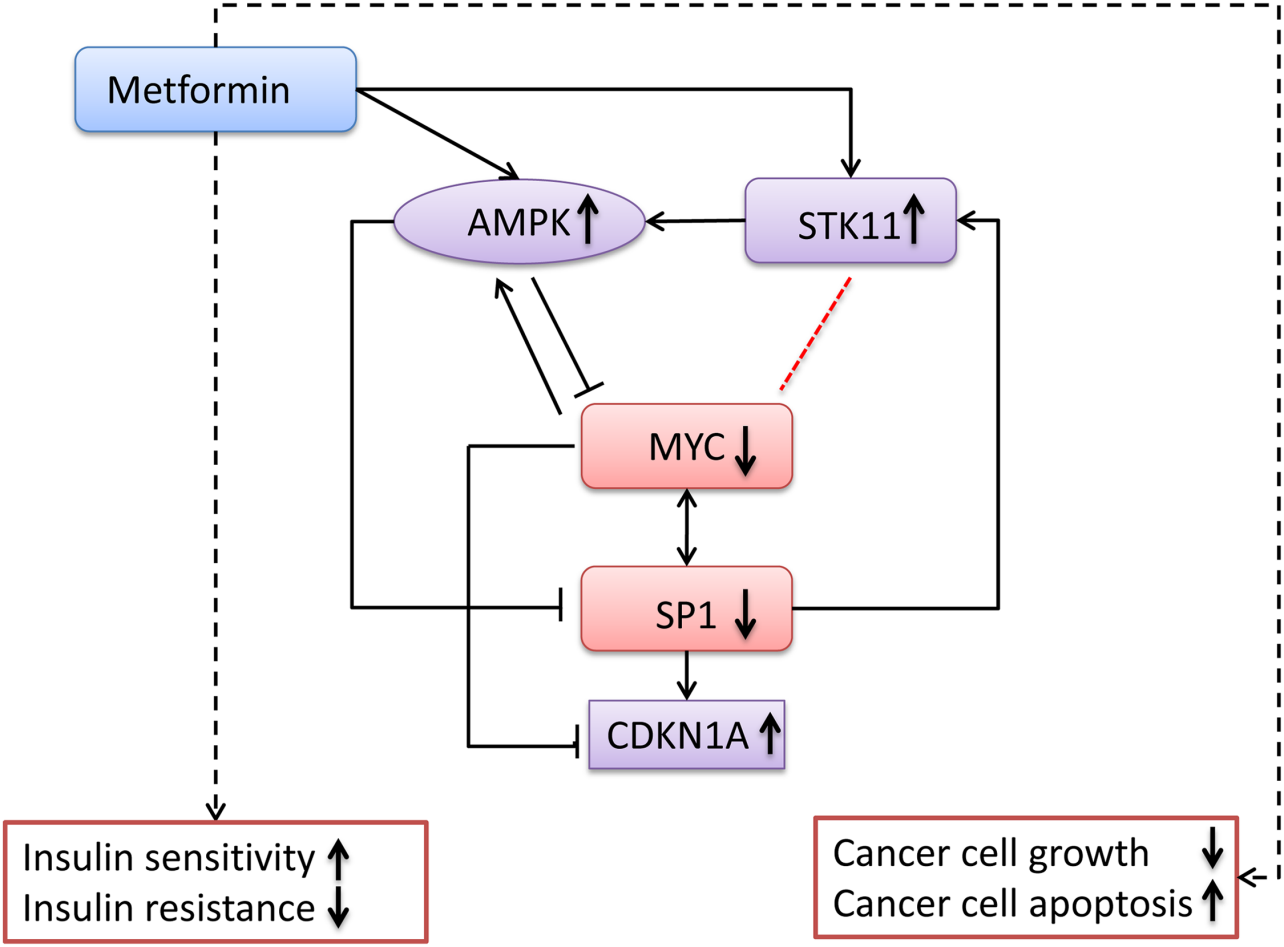
A novel metformin action pathway. Solid lines indicate the proposed mechanisms as supported by experimental evidence from literature. The two black dashed lines indicate the drug effects. The red dashed line indicates the relationship is existed but the direction is unknown. The arrows beside the gene names or biological processes indicate the metformin effects. Up-arrows indicate the corresponding genes or processes are up-regulated while the down-arrows indicate the corresponding genes or process are down-regulated.

Starting from above crosstalk subnetwork and the seven key nodes, we manually checked their publications and integrated the experimental evidence for further understanding their roles in the metformin actions. Through careful review, we summarized their function and action together and found that a novel MYC-centered pathway was hidden under the crosstalk subnetwork, which may play important roles in metformin action in T2D and cancer (Fig 7). The Myc-centered pathway included AMPK, STK11, MYC, SP1, and CDKN1A, which formed two small motifs: AMPK-STK11-MYC and MYC-SP1-CDKN1A.

It is well known that metformin exerts anti-diabetes and anti-cancer effects via mitochondrial complex I inhibition [68,69]. Mitochondrial complex I inhibition increases AMP/ATP ratio, which activates AMP-activated protein kinases (AMPKs) [70] to cause human disease [71]. In the crosstalk subnetwork, the first module contained core members of AMPK signaling pathways (PRKAA2, PRKAB2, and PRKAG2), which is linked to the second and third modules through the STK11-MYC interaction. The gene *LKB1*encodes a key upstream activator of AMPK [51] and is known to be inactivated through mutations during lung carcinogenesis [72]. Furthermore, the metformin induces activation of LKB1 [73]. For the MYC and LKB1, several lines of evidence show they are in opposite action in tumor. For example, LKB1 is overexpressed partly by degradation of MYC protein to inhibit lung carcinoma cell proliferation [74]. Nevertheless, their direct relationship is not clear. Recent studies have shown that metformin has an ability to reduce MYC protein level in vivo and in vitro in several types of cancer, including lung cancer [75] and prostate cancer [76]. Based on the integrative network and function analyses with experimental evidence, we suggested a feed-forward loop (AMPK-STK11-MYC) exists in metformin action. This network motif may act cohesively to strengthen the inhibition of MYC expression.

In addition, in the crosstalk subnetwork, three nodes (CDKN1A, MYC, and SP1) formed a 3-node clique. The network small motif bridges the three modules together. The SP1 is a TF that binds to the GC-rich motif of numerous genes’ promoters and is involved in many cellular processes, including cell differentiation, cell growth, apoptosis, immune responses, response to DNA damage, and chromatin remodeling. It has been reported that SP1 could cooperate with MYC to activate transcription of the human telomerase reverse transcriptase gene (*TERT*), which is responsible for maintenance of the length of telomeres and its defects may lead to diseases including cancer [77]. During the process of carcinogenesis, expression of *MYC* and *SP1* is known to be up-regulated [78]. It has been reported that metformin has an ability to down-regulate MYC [75,76] and SP1 [61]. Additionally, MYC [79,80] and SP1 [81,82] are also the key transcription factors involved in the regulation of insulin and insulin regulated gene transcription. MYC could directly induce both impaired insulin secretion and loss of β-cell mass [83]. SP1 could regulate the upstream target STK11 expression [84,85]. MYC could activate AMPK in multiple cell lines [86]. AMPK activation could reduce SP1 translocate from cytoplasm to nucleus [87]. The *CDKN1A*, a cyclin-dependent kinase inhibitor p21, inhibits proliferation both in vitro and in vivo. After metformin treatment, the expression of *CDKN1A* is upregulated in hepatocellular carcinoma [88] and bladder cancer cells [89]. Additionally, multiple lines of evidence have demonstrated that MYC can suppress the expression of *CDKN1A* in cancer like colorectal cancer [90]. Therefore, taken all evidence together with the crosstalk network, we propose a new biological pathway for metformin action focused on four key nodes (CKDN1A, MYC, SP1, and STK11) (Fig 7). The pathway highlights several new questions, which may have been missed by previous studies. Specifically, we speculate that MYC and its networks are the key downstream targets of metformin. Further investigations are needed to illustrate this mechanism.

## Discussion

In this study, we developed a computational framework (DSPathNet) to construct a signaling pathway network for a given drug, specifically, metformin. The framework first collected metformin upstream genes from different data sources and inferred chemical signaling receptor TFs based on metformin-induced gene expression data. Then, a metformin-specific SPNetwork was produced using the random walk-based algorithms by applying longitudinal and lateral movements starting from metformin upstream genes and downstream TFs. By examining the enrichment of disease genes in the network, the metformin-specific SPNetwork proved to be enriched with genes that could contribute to the pathology of T2D and cancer, or reducing cancer risk in T2D patients undergoing metformin treatment. Starting from the genes common to T2D and cancer GWAS data, we further produced a crosstalk subnetwork of metformin action in T2D and cancer. Through comprehensive network and functional analyses and literature mining, we identified seven critical genes (*CDKN1A*, *ESR1*, *MAX*, *MYC*, *PPARGC1A*, *STK11*, and *SP1*), some of which have been implicated in previous studies. Furthermore, the MYC and its motifs were suggested to play important roles in metformin action. In summary, this study has the following major results: 1) we developed a computational framework for building drug-specific signaling pathway networks; 2) we generated a metformin-specific signaling pathway network that is significantly enriched with genes associated with T2D, cancer, or metformin-associated cancer survival, and 3) we pinpointed the MYC-centered pathway that may play important roles in metformin action. These results demonstrate that the computational framework effectively integrates various types of data, such as prior drug knowledge and drug-induced gene expression to identify critical genetic factors responsible for drug indications and drug response. This framework is a novel approach that provided a broader and deeper understanding of metformin actions in both T2D and cancer. This computational approach can be applied to other drugs as well.

This framework applies a new network generation strategy that focused on a drug of interest. In our framework, we utilized the gene expression data to infer the drug related gene expression regulators TFs, which is different from the methods that have been developed to infer signaling pathway networks directly from gene expression data [91]. As we know, the gene expression represents the transcriptional changes in the downstream genes of a pathway and provides an indirect view of pathway structure and gene activity after modulation of the system. Thus, the gene expression cannot directly represent the activity state of many signaling components that mediated the cellular response [92]. It is well known that the signal transduction network is not linear; rather it is quite complex [3]. During the development of this framework, we observed only two genes overlapped between metformin upstream genes and downstream genes. This small overlap presents us with a big challenge: how to fill the gap to rebuild a complete cascade for drug action? To tackle this challenge, we proposed a novel strategy from background human SPNetwork through both longitudinal and lateral movements. For the longitudinal movement, we employed the software NetWalker that implemented the random walk with a starting probability. For the lateral movement, we took advantage of K-Walk algorithm that simulates random walks in the network using a Markov Chain to build the most relevant subnetwork. In this study, we combined them together to achieve our goal. Table 2 summarizes the number of genes in each step and the hypergeometric tests based on the number of genes with smallest *P*-value less than 0.05 in the corresponding network compared to all genotyping data in T2D GWAS data. The evaluation results indicated that the process is promising since it recruited more informative genes; the significance of the association between the network and disease-related mutation signals became stronger.

However, the major concern regarding the framework is to rebuild a complete and reliable human SPNetwork and to control false positives from both public data and prediction results caused by the computational tools. To balance these two factors, we rigorously compiled the information involved in the signaling pathways, extensively collected the drug related data from multiple data sources, applied rigorous parameters during the use of computational approaches, and performed comprehensive evaluations for metformin-specific SPNetwork. To increase the accuracy of results, we only included the protein-protein pairs with experimental evidence and excluded the pairs only involved in the protein complexes. Thus, the coverage of the human SPNetwork was lower than a typical protein-protein interaction network; it contained only 37,881 edges and 4,367 proteins. With the rapid development of human experimental technologies, we believe more data with higher coverage and accuracy will become available, which will enable the construction of a more comprehensive signaling pathway network with high quality. To collect as many metformin-related genes as possible, in addition to the public databases DrugBank and PharmGBK, we further performed literature mining from PubMed abstracts, which provided an additional 19 genes. To ensure the accuracy of TF inference, we only utilized gene expression data from the four treatments of metformin that showed significant consistency with each other. To comprehensively evaluate if the metformin-specific SPNetwork was enriched with mutation signals of T2D and cancer, we not only took advantage of the well-studied disease genes but also individual genotyping data from GWAS data sets. Thus, our framework has the ability to recruit more key components in the drug signal transduction process. It could be potentially applied to other drugs for the purpose of deciphering their signaling pathway networks and identifying critical genes. Another limitation of this framework is the absence of a control network representing the normal state. The signaling network at the normal state may provide additional insights into drug action. However, it is very difficult and challenging to construct a normal-state signaling transduction network for drug action. Though some pathway data sources such as KEGG provide the relevant signaling networks in the normal state, most of them only provide a limited view by focusing on one or two related pathways. Compared to these individual pathway networks, the metformin-specific SPNetwork provides a comprehensive view by including many well-known metformin-related pathways, T2D-related pathways, and cancer-related pathways (Results).

This computational framework is strongly dependent on the available literature about the investigated drugs. Thus, it is not suitable for these drugs or chemicals that do not have many basic research reports. However, it is known that, during the drug development, most of them cannot be approved by FDA even after entering the clinical trials [93]. Furthermore, as the time and costs for developing novel drugs dramatically increased recently, many drug developers prefer to find new uses for existing drugs including the approved and non-approved drugs. As more large-scale data become publicly available, researchers could utilize the framework to build a SPNetwork for each drug of interest, and then examine the relationship between the network and disease genes, or calculate network similarities with the known drugs for a certain indication. These relationship or network similarities may provide more clues for drug repurposing at the network level. Therefore, the framework will be promising for identification of drugs that may be used to treat secondary indications by constructing and comparing the drug-specific SPNetworks. Moreover, since the drug-specific SPNetwork contains comprehensive information regarding the drug action of the components, we speculated that some off-targets might be included in the network. Thus, our network approach can be extended to evaluate the association between drugs and their potential side effects. However, it is challenging to identify large-scale side effect data associated with genes or their proteins. So far, several studies have used the available biochemical data to determine candidate targets for specific side effects [94–96]. Such data is limited and likely with a high false positive rate. When more relevant data becomes available in future, our approach will be applied to assess drugs’ side effects.

An important output of this study is the metformin-specific SPNetwork consisted of metformin related genes, metformin related TFs, and many novel genes. The network provides a valuable gene pool for further investigation of metformin action. Metformin has been used to treat diabetic disorders for many years because of its ability to lower glucose levels and improve insulin sensitivity [97]. Recently, several findings from epidemiological studies have shown that metformin can reduce cancer risk and improve cancer survival in the T2D patients [60,98,99], including a recent electronic health record (EHR) study we participated in that demonstrated the effect was seen for many cancer types [26]. However, the molecular mechanisms underlying metformin action are complex and remain unclear, especially for its ability of decreased cancer risk [100,101]. In this study, we first constructed a complex metformin-specific SPNetwork and then produced a crosstalk subnetwork from the metformin-specific SPNetwork. This subnetwork contained three modules highlighting different pathways (Fig 6C). The first and second modules were enriched with genes from the insulin signaling pathway and adipocytokine signaling pathway, and the third module was enriched with genes involved in cancer related pathways. The adipocytokine signaling pathway contains the major components of AMPK signaling pathway according to KEGG annotation. Through seven nodes, the first and second modules were linked to the third module. These observations suggest that the metformin possibly affects the AMPK signaling pathway and the insulin signaling pathway directly, which subsequently decrease the chance of cancer development. This outlook is consistent with a previous review [102].

The seven nodes act as bridges linking the first and second modules to the third module. We predicted they might play critical roles in the metformin signaling transduction process (Fig 6C). Among them, two genes (*PPARGC1A* and *STK11*) belonged to metformin upstream genes; one (*ESR1*) to metformin downstream genes; four genes (*CDKN1A*, *MAX*, *MYC*, and *SP1*) were both hubs and bridge nodes. It is well known that gene *STK11*, also known as *LKB1*, encodes a member of the serine/threonine kinase family that regulates cell polarity and functions as a tumor suppressor [103]. Additionally, previous studies have shown that mutations in the *STK11* gene influence insulin sensitivity and metformin efficacy [104,105]. The *MYC* gene encodes a protein that plays a role in cell cycle progression, apoptosis, and cellular transformation [106]. It has been shown that *MYC* gene plays important roles in the anticancer metabolic effects of metformin [75,76]. The *PPARGC1A* gene encodes a transcriptional coactivator that regulates the genes involved in energy metabolism. Its variant rs2970852 has been reported to modify the effects of metformin on triacylglycerol levels [107]. Recent studies have shown that gene regulation induced by metformin involves the transcription factor SP1 in cancers [61,108]. Moreover, the expression of *CDKN1A* (also known as *P21*) is upregulated in hepatocellular carcinoma [88] and bladder cancer cells [89] after metformin treatment. The evidence from these studies suggests that our approach is effective for identifying the key components in the signaling pathway. To further investigate detailed information for these genes, more experimental validations are needed. To our knowledge, there is no any positive evidence for the association of the genes *ESR1* and *MAX* of the seven critical genes with metformin action. Thus, they are two novel genes for further experimental validation.

In addition to the DSPathNet framework to effectively recruit critical components in the mode of drug action, there are other ways to expand this approach. First, integrating multiple layers of data involving the signal cascade beyond gene expression data into a comprehensive method might improve our ability to identify the association between the genetic changes and their response to drugs. Second, although we have shown the utility of two sources for compiling the human SPNetwork, there are other data worth exploring such as those involved in the metabolism, protein phosphorylation, and protein kinase and phosphatase interactions. While this study focused on one medication, metformin, the computational framework is broadly applicable to any drug for which induced gene expression data is available. Moreover, several experimental data sources are available for further data integration and mining such as the Connectivity Map project [15], Genomics of Drug Sensitivity in Cancer [109], Cancer Cell Line Encyclopedia (CCLE) [110], and anticancer compounds in breast cancer [111]. Finally, analyzing the crosstalk among different types of diseases in the context of networks will offer an intriguing opportunity to explore the underlying molecular mechanisms of drug action, which will provide an alternative approach for drug repurposing.

## Materials and Methods

### Compilation of one human SPNetwork with weighted nodes

Before generating the metformin-specific SPNetwork, we need a global signal transduction network for humans as the background network. We therefore integrated signaling transduction related associations from Pathway Commons with experimental evidence [25], and TF-TF/target pairs from TRANSFAC [26]. The Pathway Commons database collected publicly available pathways from multiple organisms with over 1,400 pathways and 687,000 interactions. We first downloaded the edge data specific for humans from the Pathway Commons (release 2011.10). Since the interactions that occur within the protein complexes do not reveal the flow of signaling information [3], we excluded the edges that came from the same complex. This process resulted in 33,614 pairs among 3,502 proteins. Additionally, we obtained 1,325 pairs among 487 TFs, and 2,723 pairs between 428 TFs and 1,315 targets downloaded from TRANSFAC database (release 2011.4). The TRANSFAC database manually collects eukaryotic TFs, their genomic binding sites, and DNA binding profiles with experimental evidence [112]. After merging the two data sets and removing the redundancies, we obtained a network with 37,881 edges and 4,367 nodes. This network was used to represent global signaling pathways in humans.

To further weight the association of each node in human SPNetwork with metformin action, we assigned a functional similarity score by calculating its functional similarity to the metformin upstream genes using the R package GoSemSim based on GO annotations [113]. GO annotations have three functional domains (*k*): molecular function (MF), biological process (BP), and cellular component (CC). First, for a given node *i* in each domain (*k*), we calculated its score as ${Score}_{i}\;=\;\sum_{j\;=\;1}^{n}{Score}_{i,j}/n$, where *n* is the number of existing scores between node *i* and metformin upstream gene *j*. Second, for the given node *i* in all domains, we calculated a final score as $\hat{S}\;=\;\sum_{k\;=\;1}^{N}{Score}_{k}/N,$ where *N* is the number of the domains having scores for the node.

### Inference of metformin downstream genes

Gene expression profiles of cancer cells following drug treatment are useful for better understanding cellular changes reflective of drug treatment [114]. In this study, we integrated the known TF-target association and drug-induced gene expression data to infer the metformin downstreams. We first comprehensively collected the TF-target associations, then calculated the up- or down-regulated genes from drug-induced gene expression data, and finally performed the hypergeometric test to evaluate the over-representation of the up- or down-regulated genes in multiple TF target gene datasets.

To compile a target gene set for each TF comprehensively, we downloaded data from two sources: TRANSFAC Professional (release 2011.4) and MSigDB database [38]. From the TRANSFAC database, we extracted known TFs and their targets in human. From the MSigDB, we downloaded the gene sets that share one TF binding site. The gene sets were derived from a comparative analysis of human, mouse, rat, and dog genomes and were organized by TF binding motifs. Genes associated with different binding motifs that correspond to a common transcription factor were combined into one gene set. After merging the two data sets, we obtained 666 human TFs and 8,502 human targets.

To calculate the potential differentially expressed genes induced by metformin, we downloaded ten gene expression datasets from Connectivity Map website (version 2.0). The gene expression datasets were generated from metformin treated cell lines. We calculated the ranked probes by using the method described in Lamb et al. [15] and selected the top 100 and bottom 100 probes in each treatment to represent the differentially expressed probes [115]. We examined the expression consistency among them using the software GSEA. We noticed that, among ten metformin treatment data sets, four had the highest consistency based on GSEA analysis [38]. Therefore, we utilized these four treatment gene expression data to perform the GSEA leading edge analysis to detect differentially expressed probes. Then, by mapping the differently expressed probes to genes using Ingenuity Pathway Analysis Tool (http://www.ingenuity.com/), we obtained the up-regulated genes and down-regulated genes.

Finally, we performed the hypergeometric test to evaluate the over-representation of the up- or down-regulated genes in the different TF gene sets. The TFs with *P*-value less than 0.05 were identified as significant TFs related to metformin action and their genes as metformin downstream genes.

### Construction of metformin-specific SPNetwork

Considering that the signal transduction cascade is not linear, we adopted a two-step strategy to construct the metformin-specific SPNetwork from the human SPNetwork. More specifically, in the first step, we utilized the software NetWalker to expand metformin upstream genes and downstream genes for longitudinal conduction [116]. The NetWalker implements the random walk with a starting probability. In this study, we gave equal starting probability of 0.5 to each gene in the metformin upstream genes and downstream genes and required those nodes with both local *P*-value < 0.05 and global *P*-value < 0.05 as the expanded genes. In the second step, we expanded the nodes from in the first step by lateral movement by applying the K-Walk method implemented in the Python package GenRev [117]. The K-Walk algorithm simulates random walks in the network using a Markov Chain to build the most relevant subnetwork, connecting seed nodes by walk a fixed length *L* or up to a maximal length *Lmax* in a large network. A subnetwork is obtained by keeping only edges that are above a minimal relevance threshold. The threshold is automatically fixed after the subnetwork has the maximum score. As such, the limited K-Walk algorithm computes edge and node relevance from random walks connecting the seed nodes [118].

### GWAS data sets

We used one T2D GWAS data set, three cancer GWAS data sets, and one GWAS data set for T2D patients with metformin treatment. The T2D GWAS data was individual-level genotype data generated from the WTCCC [31]. The three cancer GWAS datasets were generated by the Cancer Genetic Markers of Susceptibility (CGEMS) project: breast cancer [32], pancreatic cancer [33], and prostate cancer [32]. We downloaded the genotype data from the National Center for Biotechnology Information (NCBI) dbGaP with approved access for the CGEMS project. For these four GWAS datasets, we first removed individuals with genotyping rate < 95% and SNPs with missing rate >5%. A single SNP associated test was conducted using the Armitage trend test for SNPs with a minor allele frequency (MAF) > 0.05. S10 Table summarizes the data.

T2D cancer patients from Vanderbilt University Medical Center (VUMC) were identified using the Synthetic Derivative (SD), a de-identified copy of the electronic health records from VUMC. Eligible subjects were individuals who 1) had a cancer diagnosis (excluding non-melanoma skin cancers) between January 1, 1995 and December 31, 2010 identified through the Vanderbilt tumor registry, and 2) were older than 18 years at the time of cancer diagnosis. Using a previously developed algorithm [119,120], we identified T2D subjects having at least two pieces of clinical information in their medical record: 1) ICD9 code for type 2 diabetes, 2) medications for type 2 diabetes, or 3) clinical labs suggestive of T2D (random glucose >200 mg/dl or hemoglobin A1c > 6.5%). Individuals without at least two of the above types of information were excluded. At least two mentions of metformin use (mono-therapy or combined therapeutic) and one mention of metformin use within 5 years after cancer diagnosis were required for study inclusion. Individuals on other T2D medications were excluded from analysis. Subjects were followed for overall mortality that was determined through linkage with the Vanderbilt tumor registry. Physician-reported European descent individuals with an available DNA sample in the Vanderbilt biobank (BioVU) [121] were genotyped on either the Illumina HumanOmni1-Quad or the Illumina HumanOmni5-Quad. Only the consensus single nucleotide polymorphisms (SNPs) between the two genotyping platforms were used. Standard quality control (QC) procedures were applied to remove individuals and autosomal SNPs not meeting standard QC criteria (i.e. related individuals, discordant sex, sample efficiency < 98%, genotyping efficiency < 98%, deviations from Hardy-Weinberg equilibrium (p < 1×10^–6^), and MAF < 5%). Palindromic SNPs were also removed. After QC, 461 individuals and 551,745 SNPs remained. Principal components were estimated using EIGENSTRAT [122]. The association between each SNP, assuming an additive genetic model, and overall survival was examined using Cox proportional hazards models, adjusted for age, sex and one principal component, using the GenABLE package of R [123]. The GWAS analysis of this set is ongoing and will be reported in a separate publication.

In this study, we defined the genes having at least one SNP with nominal *P*-value less than 0.05 as disease or drug related genes. The SNP is located in the gene’s region or its 20kb up- or down-stream sequence based on the gene annotation and human reference genome build 36 for T2D GWAS study and cancer GWAS studies and build 37 for metformin GWAS study.

### Pathway enrichment, network analysis and visualization

To identify pathways overrepresented in gene sets, we performed KEGG pathway enrichment analyses using WebGestalt [49] (version 1/30/2013). Given a list of genes, a hypergeometric test was performed for the enrichment of these genes, which was implemented in the WebGestalt tool. To control the error rate in the analysis results, WebGestalt also provides a corrected *P*-value based on the Benjamini-Hochberg method [124]. To summarize the enriched pathways, we took advantage of KEGG pathway category annotation, which included the two-level categories and represent the relative abundance of the pathways [125]. These pathways are grouped into seven categories at the first level of KEGG annotation and 43 categories at the second level of KEGG annotation. At the second-level category, we further calculated a Z-score for each category to represent the KEGG pathway relative abundance: Z-score = $\frac{x-u}{\sigma }$, where *x* is the number of pathways in one category in the first or second level, *u* is the mean of the pathway number in the first or second category, *σ* is the standard deviation of the pathway number in the first or second category. The pathway categories were selected for further analysis if their Z-scores were higher than zero.

In this study, we adopted the statistical design for gene set enrichment analysis [126] to compare a gene set (A) in the drug-specific network to a reference gene set (B). The design has been commonly used to conduct the gene annotation enrichment analysis [127]. Suppose that the gene set (A) has *n* genes, of which most genes (*n’*) belong to the reference gene set (*m*). Among *n’* gene, *k* genes belong to a given category (C). And the reference gene set has *j* genes belong to the same category (C). Based on the definition of the hypergeometric test, we performed the hypergeometric test to get a *P*-value to evaluate the significance of enrichment for category C in the gene set A.

For network property analysis, we calculated degree of each node and degree distribution of all nodes, which are the most basic measures of biological networks [41]. The node degree (connectivity) is the number of links of a node in the network. If degree distribution of one network follows a power law, the network would have only a small portion of nodes with a large number of links (i.e., hubs) [41]. To determine the hubs in metformin-specific SPNetwork, we adopted the method utilized by Yu et al. [46], as we did in a previous study. We first drew a degree distribution for the whole network to define a specific degree value as a cut-off point (S12 Fig). If a node has the degree greater than the cut-off value, then the node is a hub. To identify the modules, we performed the cluster and community analysis using the software CFinder (version 2.0.5) [63]. CFinder is a fast program to locate and visualize overlapping, densely interconnected groups of nodes in undirected network. We required each node in the module being involved in at least one 3-vertex clique. We visualized the networks using Cytoscape (version 3.2) [128].

## Supporting Information

S1 Fig The three-way Venn diagram summarizes the number of shared genes among the three gene sets. The “Human SPNetwork node” represents the genes corresponding to nodes in the human SPNetwork, ‘Gene_46’ represents the metformin-related genes obtained from DrugBank and PharmGKB, and ‘Gene_29’ represents the metformin-related gene obtained by literature searching approach.
(TIFF)

S2 Fig GSEA (Gene Set Enrichment Analysis) enrichment score curve for six probe sets of six treatments (Instance IDs: 61, 1694, 1816, 1858, 5068, and 5487) compared to the probes from one treatment (Instance ID: 1). In each graph, the vertical black lines indicate the position of each of the probes of the studied probe set in the ordered, non-redundant data set. The green curve corresponds to the ES (enrichment score) curve, which is the running sum of the weighted enrichment score in GSEA.
(PDF)

S3 Fig Network of metformin upstream gene and downstream genes. This network was generated by mapping them129 unique genes of metformin upstream genes and TF genes to human SPNetwork. The nodes and edges in orange correspond to nodes and edges only in the metformin upstream network. The nodes and edges in green correspond to the nodes and edges only in the metformin downstream network. And the nodes and edges in red correspond to the nodes and edges common to the metformin upstream network and the metformin downstream network.
(PNG)

S4 Fig Summary of genes by longitudinal and lateral movements from metformin upstream genes and downstream genes via three-way Venn diagrams. A) Summary of the number of shared genes among metformin upstream genes represented by ‘Upstream gene’, the genes obtained by longitudinal movement represented by ‘Longitudinal gene’ based on ‘Upstream gene’, and the genes obtained by lateral movement based on ‘Longitudinal gene’. B) Summary of the number of shared genes among metformin downstream genes represented by ‘Downstream gene’, the genes obtained by longitudinal movement represented by ‘Longitudinal gene’ based on ‘Downstream gene’, and the genes obtained by lateral movement based on ‘Longitudinal gene’.
(PNG)

S5 Fig Network of extended genes of metformin upstream genes and downstream genes by longitudinal movement. The network was generated by mapping the unique 219 genes of extended genes of metformin upstream gene and downstream genes by longitudinal moving to the human SP Network. The legends for orange nodes, red nodes, and green nodes are same as in S3 Fig.
(PNG)

S6 Fig *P*-value distribution of metformin GWAS data of the metformin-specific SPNetwork, human SPNetwork, and metformin GWAS. The details of the data were provided in Materials and Methods section.
(PNG)

S7 Fig The subnetwork for 81 genes. The genes were common to the 169 genes whose smallest *P*-values were less than 0.05 in T2D GWAS data and the 177 genes had at least one SNP with *P*-value less than 0.05 in metformin GWAS data. The legends for orange nodes, red nodes, and green nodes are same as in S3 Fig.
(PNG)

S8 Fig The subnetwork for 25 genes. These genes were common among the 169 genes whose smallest *P*-values were less than 0.05 in T2D GWAS data, 157 genes whose smallest *P*-values were less than 0.05 in breast cancer WAS data, 170 genes whose smallest *P*-values were less than 0.05 in pancreatic cancer GWAS data, 172 genes whose smallest *P*-values were less than 0.05 in prostate cancer GWAS data. The legends for orange nodes and edges, red nodes and edges, and green nodes and edges are same as in S3 Fig.
(PNG)

S9 Fig The subnetwork for 25 common genes and their direct interactors. The 25 common genes that were among the T2D GWA study and the three cancer GWA studies. The legends for orange nodes and edges, red nodes and edges, and green nodes and edges are same as in S3 Fig.
(PNG)

S10 Fig The networks for three modules. The legends for orange nodes and edges, red nodes and edges, and green nodes and edges are same as in S3 Fig.
(PNG)

S11 Fig Seven highlighted nodes in yellow in the subnetwork for 25 common genes and their direct interactors (A) and three 3-clique communities after removing the highlighted nodes (B). The legends for orange nodes and edges, red nodes and edges, and green nodes and edges are same as in S3 Fig.
(TIFF)

S12 Fig Degree distribution of the 477 nodes in metformin-specific SPNetwork. This distribution is used for determination of hubs.
(TIFF)

S1 Table Summary of data sources, software, and evaluation data used in the study. (DOCX)

S2 Table Metformin upstream genes and their sources. (DOCX)

S3 Table List of metformin treatments from Connectivity Map database. (DOCX)

S4 Table Metformin downstream genes encoding transcription factors inferred from metformin-induced gene expression data from Connectivity Map. (DOCX)

S5 Table Pairs of metformin-specific signaling pathway network (SPNetwork). (XLSX)

S6 Table List of genes in the metformin-specific SPNetwork. (XLSX)

S7 Table KEGG pathways overrepresented in 477 genes in metformin-specific SPNetwork. (XLSX)

S8 Table KEGG pathways overrepresented in upstream genes (174) only belonging to metformin upstream network, downstream genes (262) only belonging to metformin downstream network, and genes (41) common to metformin upstream network and downstream network. (XLSX)

S9 Table First-level and secondary level categories of the KEGG pathway overrepresented in upstream genes (174) only belonging to metformin upstream network, downstream genes (262) only belonging to metformin downstream network, and genes (41) common to metformin upstream network and downstream network. (XLSX)

S10 Table Summary of three cancer GWAS data. (DOCX)
