# Correction: Deciphering Signaling Pathway Networks to Understand the Molecular Mechanisms of Metformin Action

**DOI:** 10.1371/journal.pcbi.1007141

**Published:** 2019-06-14

**Authors:** The PLOS Computational Biology Staff

## Notice of Republication

This article was republished on July 29, 2015, to correct duplication in the Supporting Information that was introduced during the typesetting process. The correct version of S11 Fig has been provided and the publisher apologizes for the errors. Please download this article again to view the correct version.

## Supporting information

S1 File Republished, corrected article. (PDF)
