# Supplementary figures and images for: Deciphering Signaling Pathway Networks to Understand the Molecular Mechanisms of Metformin Action
Source: PLoS Comput Biol. 2015 Jun 17;11(6):e1004202. doi: 10.1371/journal.pcbi.1004202 (PMC4470683; doi:10.1371/journal.pcbi.1004202)

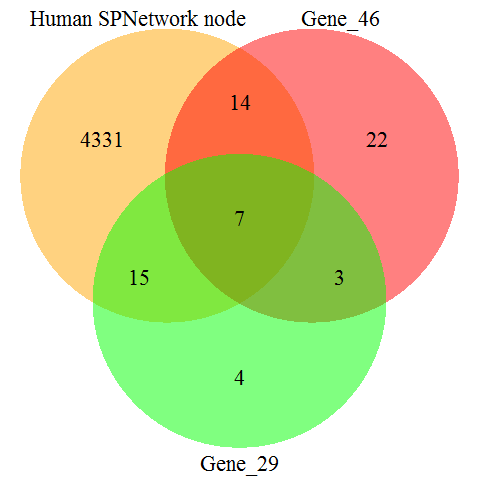

Supplement: S1 Fig — The “Human SPNetwork node” represents the genes corresponding to nodes in the human SPNetwork, ‘Gene_46’ represents the metformin-related genes obtained from DrugBank and PharmGKB, and ‘Gene_29’ represents the metformin-related gene obtained by literature searching approach. (TIFF) [file pcbi.1004202.s001.tiff]

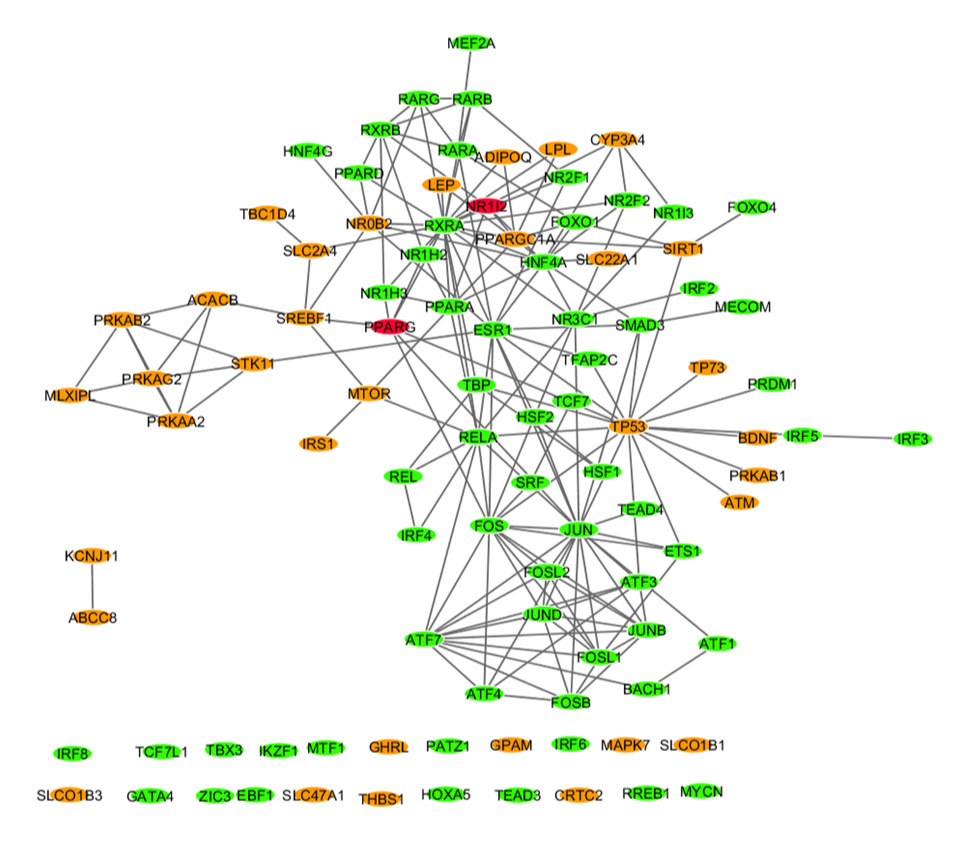

Supplement: S3 Fig — This network was generated by mapping them129 unique genes of metformin upstream genes and TF genes to human SPNetwork. The nodes and edges in orange correspond to nodes and edges only in the metformin upstream network. The nodes and edges in green correspond to the nodes and edges only in the metformin downstream network. And the nodes and edges in red correspond to the nodes and edges common to the metformin upstream network and the metformin downstream network. (PNG) [file pcbi.1004202.s003.png]

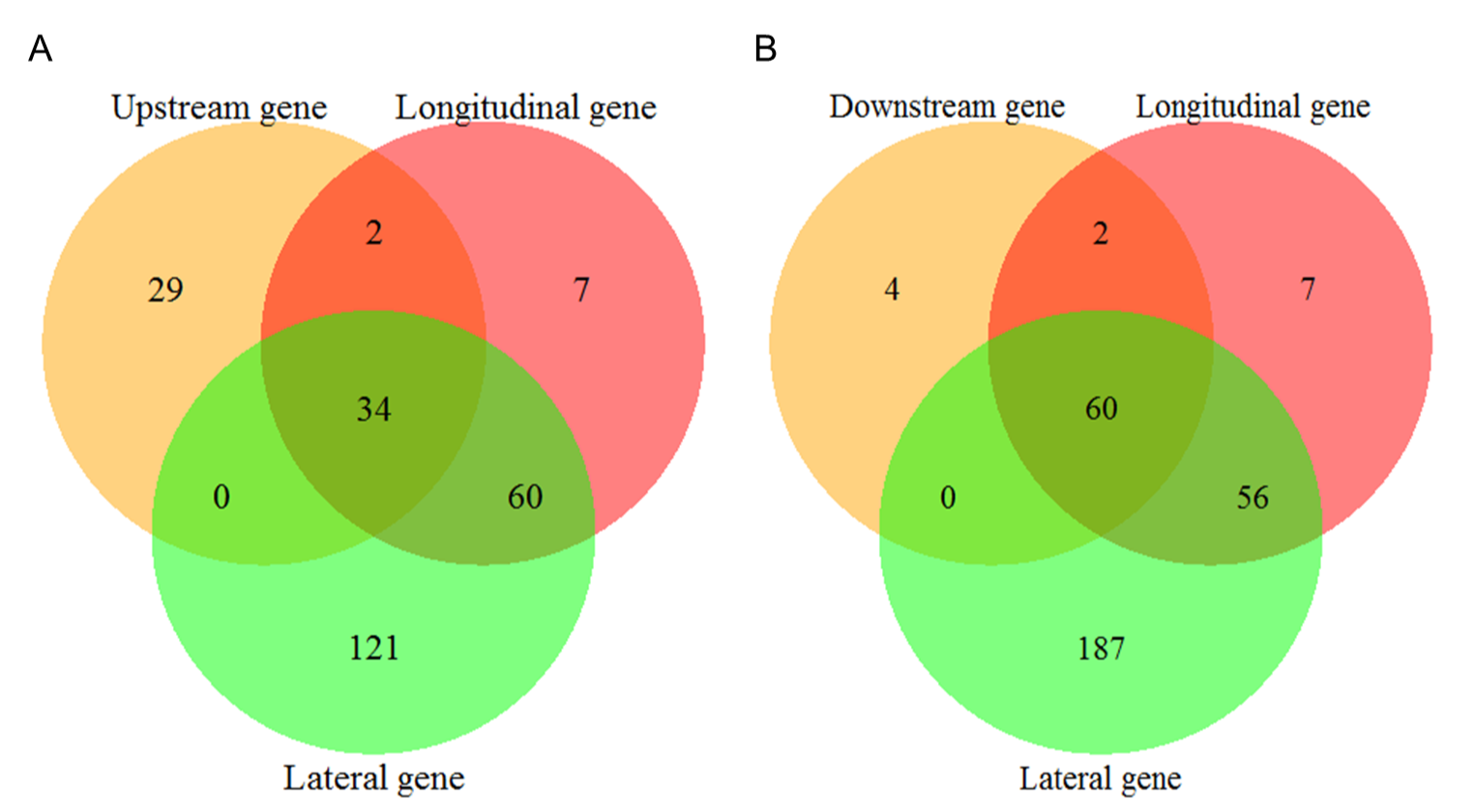

Supplement: S4 Fig — A) Summary of the number of shared genes among metformin upstream genes represented by ‘Upstream gene’, the genes obtained by longitudinal movement represented by ‘Longitudinal gene’ based on ‘Upstream gene’, and the genes obtained by lateral movement based on ‘Longitudinal gene’. B) Summary of the number of shared genes among metformin downstream genes represented by ‘Downstream gene’, the genes obtained by longitudinal movement represented by ‘Longitudinal gene’ based on ‘Downstream gene’, and the genes obtained by lateral movement based on ‘Longitudinal gene’. (PNG) [file pcbi.1004202.s004.png]

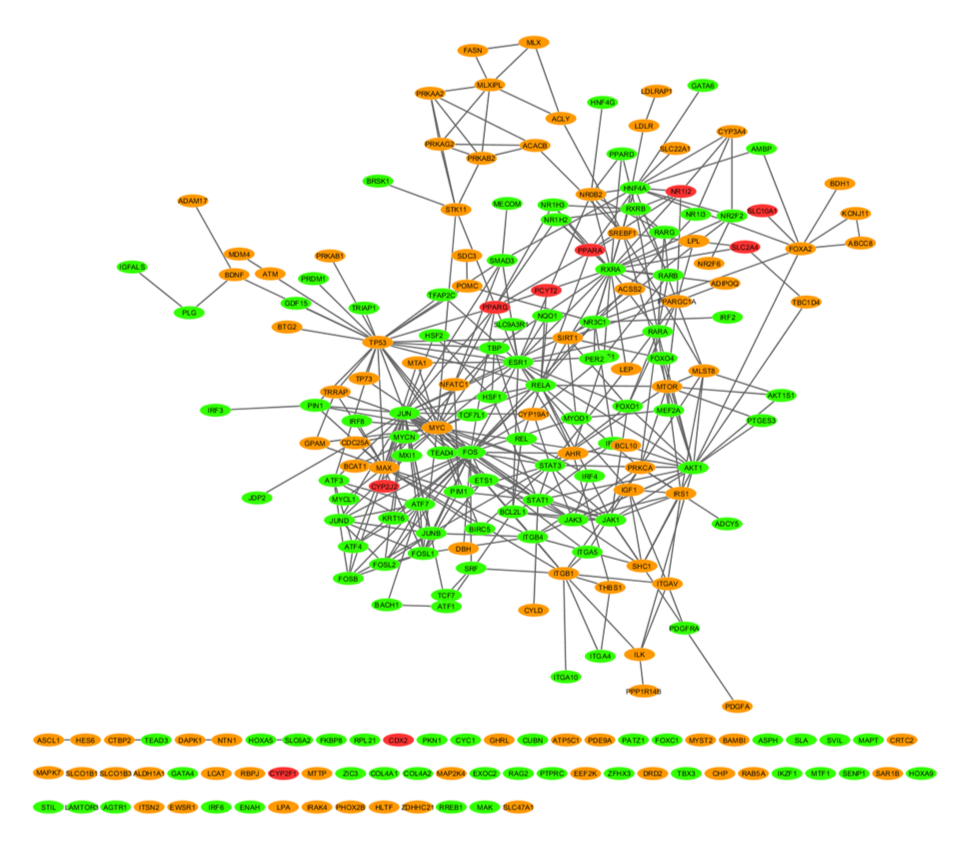

Supplement: S5 Fig — The network was generated by mapping the unique 219 genes of extended genes of metformin upstream gene and downstream genes by longitudinal moving to the human SP Network. The legends for orange nodes, red nodes, and green nodes are same as in S3 Fig. (PNG) [file pcbi.1004202.s005.png]

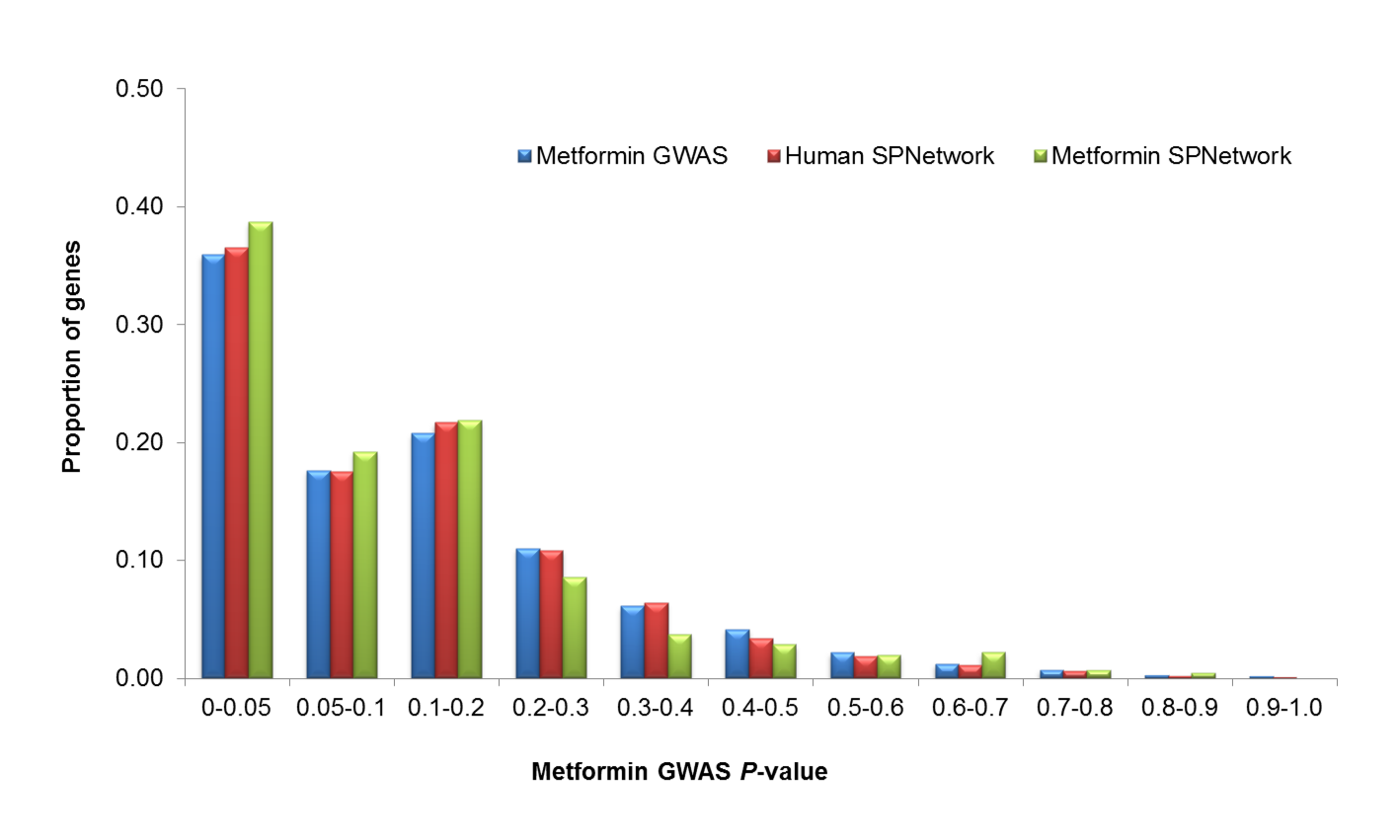

Supplement: S6 Fig — The details of the data were provided in Materials and Methods section. (PNG) [file pcbi.1004202.s006.png]

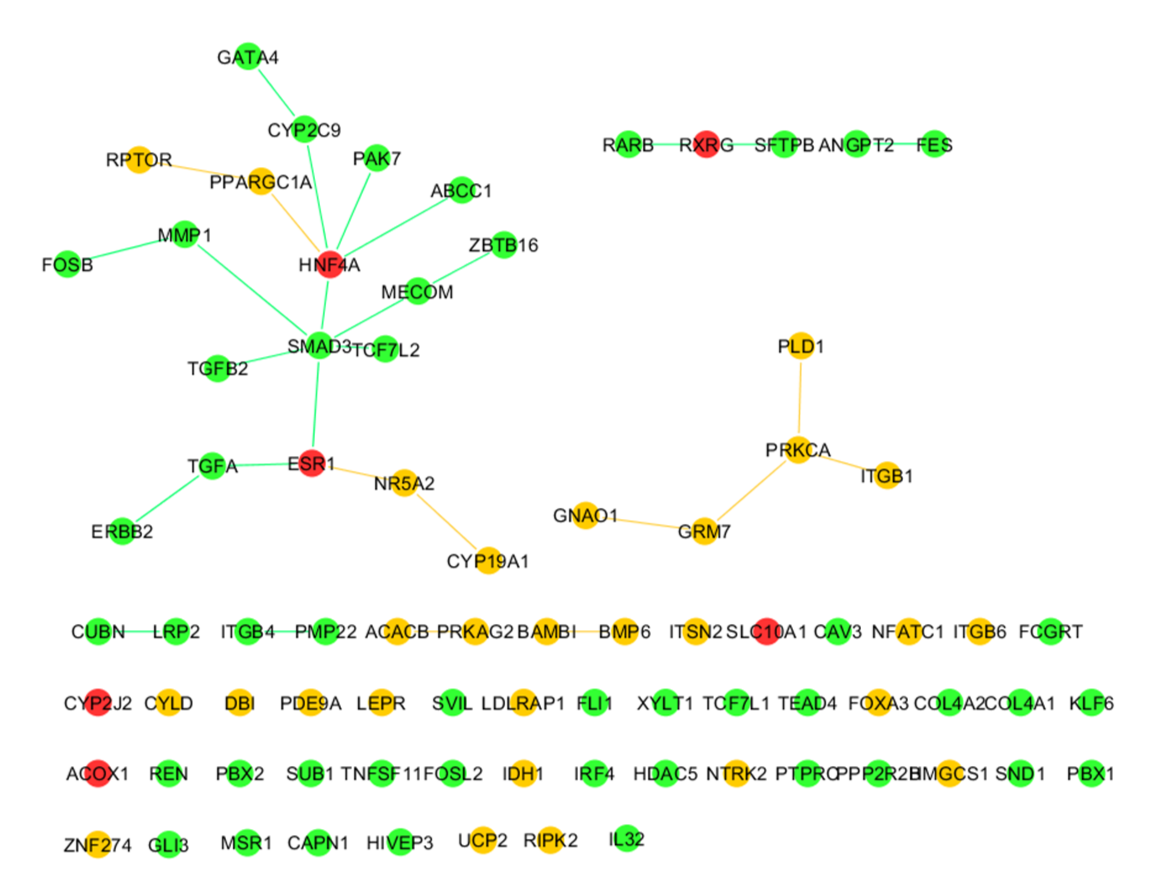

Supplement: S7 Fig — The genes were common to the 169 genes whose smallest P-values were less than 0.05 in T2D GWAS data and the 177 genes had at least one SNP with P-value less than 0.05 in metformin GWAS data. The legends for orange nodes, red nodes, and green nodes are same as in S3 Fig. (PNG) [file pcbi.1004202.s007.png]

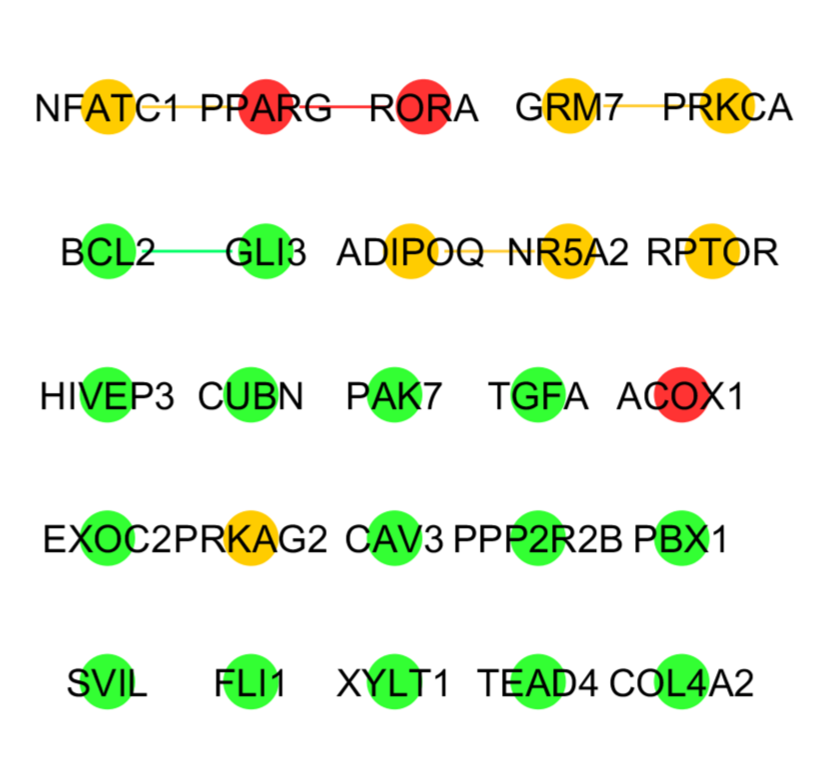

Supplement: S8 Fig — These genes were common among the 169 genes whose smallest P-values were less than 0.05 in T2D GWAS data, 157 genes whose smallest P-values were less than 0.05 in breast cancer WAS data, 170 genes whose smallest P-values were less than 0.05 in pancreatic cancer GWAS data, 172 genes whose smallest P-values were less than 0.05 in prostate cancer GWAS data. The legends for orange nodes and edges, red nodes and edges, and green nodes and edges are same as in S3 Fig. (PNG) [file pcbi.1004202.s008.png]

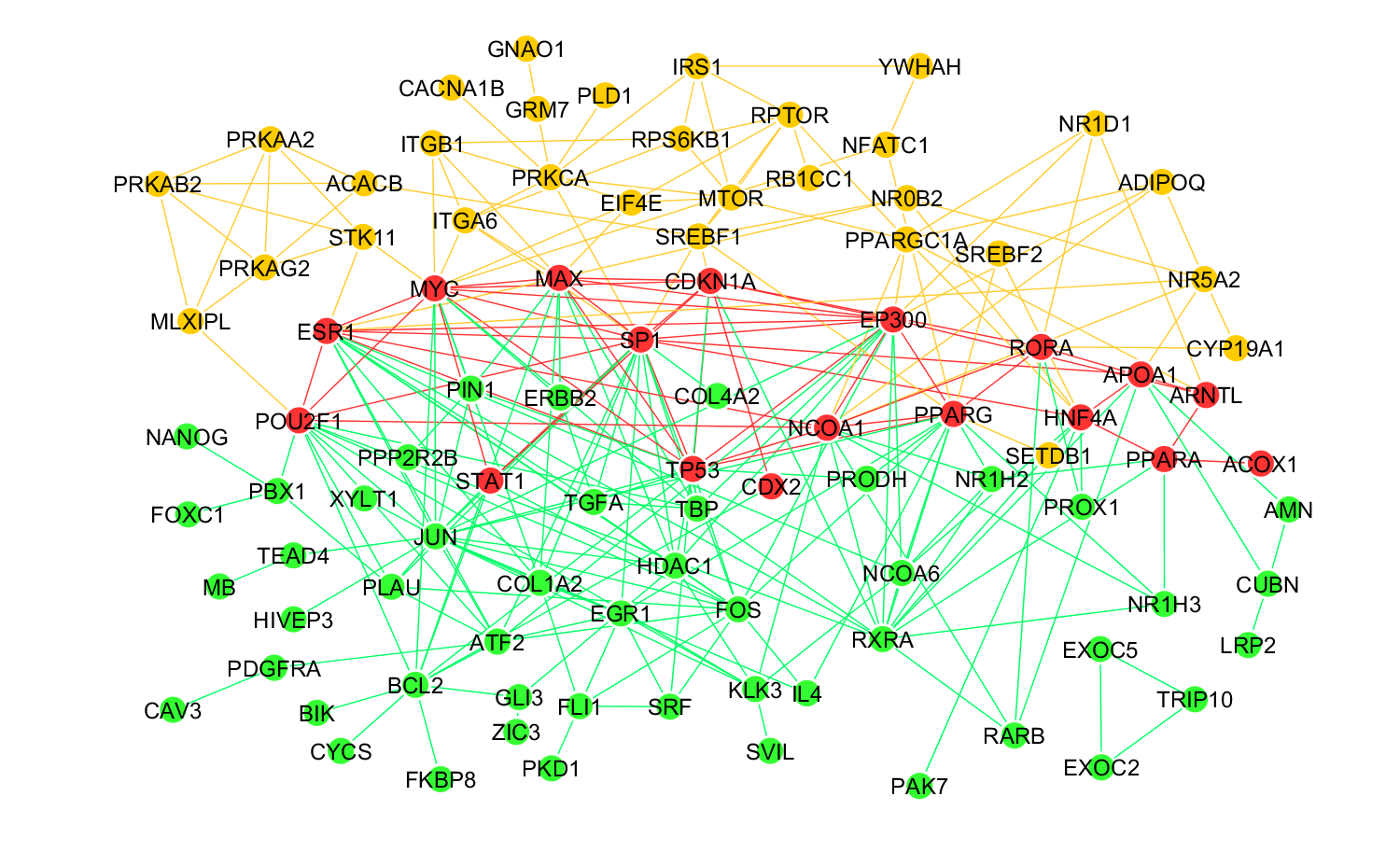

Supplement: S9 Fig — The 25 common genes that were among the T2D GWA study and the three cancer GWA studies. The legends for orange nodes and edges, red nodes and edges, and green nodes and edges are same as in S3 Fig. (PNG) [file pcbi.1004202.s009.png]

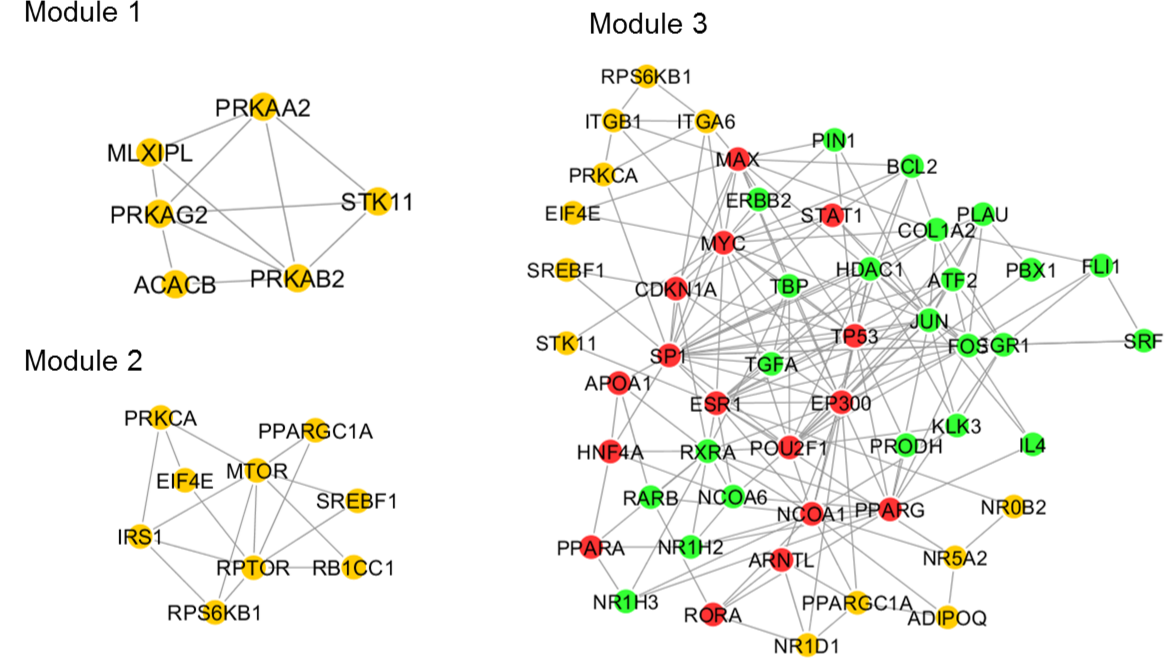

Supplement: S10 Fig — The legends for orange nodes and edges, red nodes and edges, and green nodes and edges are same as in S3 Fig. (PNG) [file pcbi.1004202.s010.png]

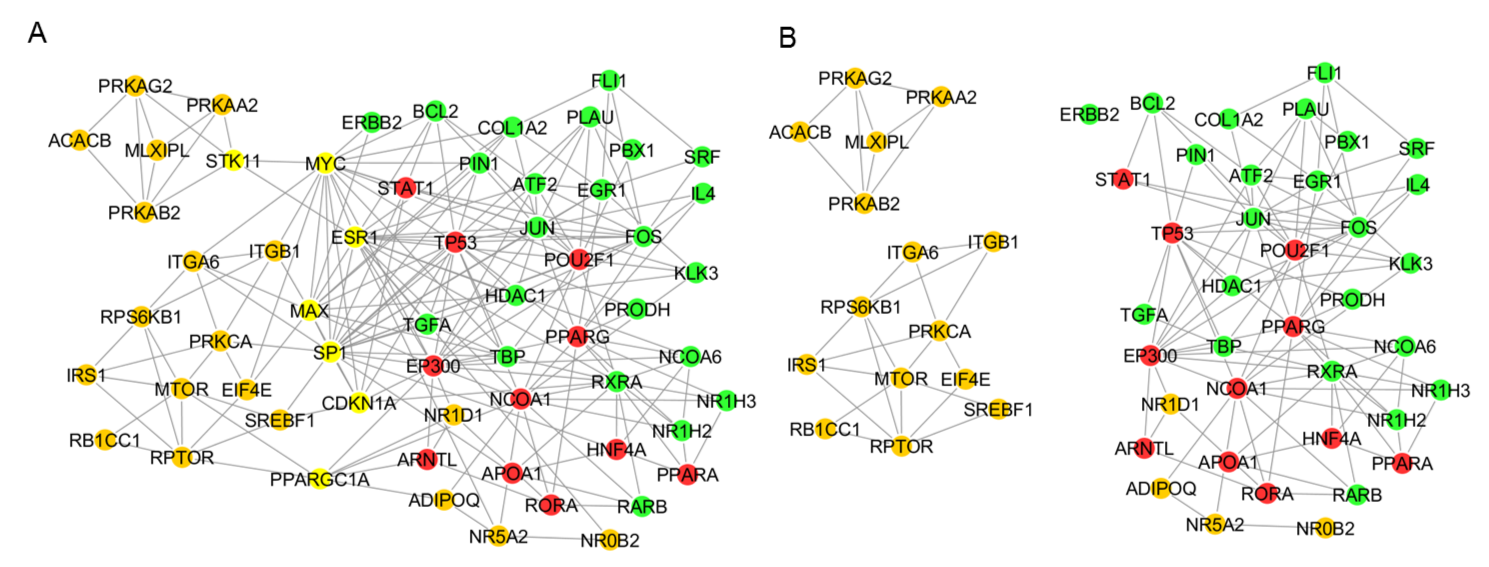

Supplement: S11 Fig — Seven highlighted nodes in yellow in the subnetwork for 25 common genes and their direct interactors (A) and three 3-clique communities after removing the highlighted nodes (B). The legends for orange nodes and edges, red nodes and edges, and green nodes and edges are same as in S3 Fig. (TIFF) [file pcbi.1004202.s011.tiff]

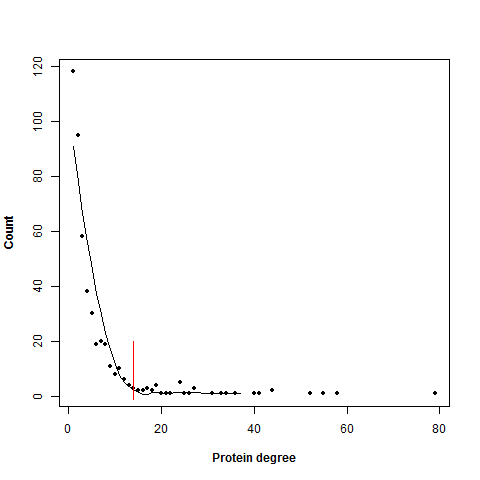

Supplement: S12 Fig — This distribution is used for determination of hubs. (TIFF) [file pcbi.1004202.s012.tiff]
