# Supplementary material for: Deciphering Signaling Pathway Networks to Understand the Molecular Mechanisms of Metformin Action
Source: PLoS Comput Biol. 2015 Jun 17;11(6):e1004202. doi: 10.1371/journal.pcbi.1004202 (PMC4470683; doi:10.1371/journal.pcbi.1004202)

Figure S2. Gene Set Enrichment Analysis (GSEA) enrichment score curve for six probe sets (Instance IDs: 61, 1694, 1816, 1858, 5068, and 5487) compared to the probes from one treatment (Instance ID: 1). In each graph, the vertical black lines indicate the position of each of the probes of the studied probe set in the ordered, non-redundant data set. The green curve corresponds to the ES (enrichment score) curve, which is the running sum of the weighted enrichment score obtained from GSEA software.

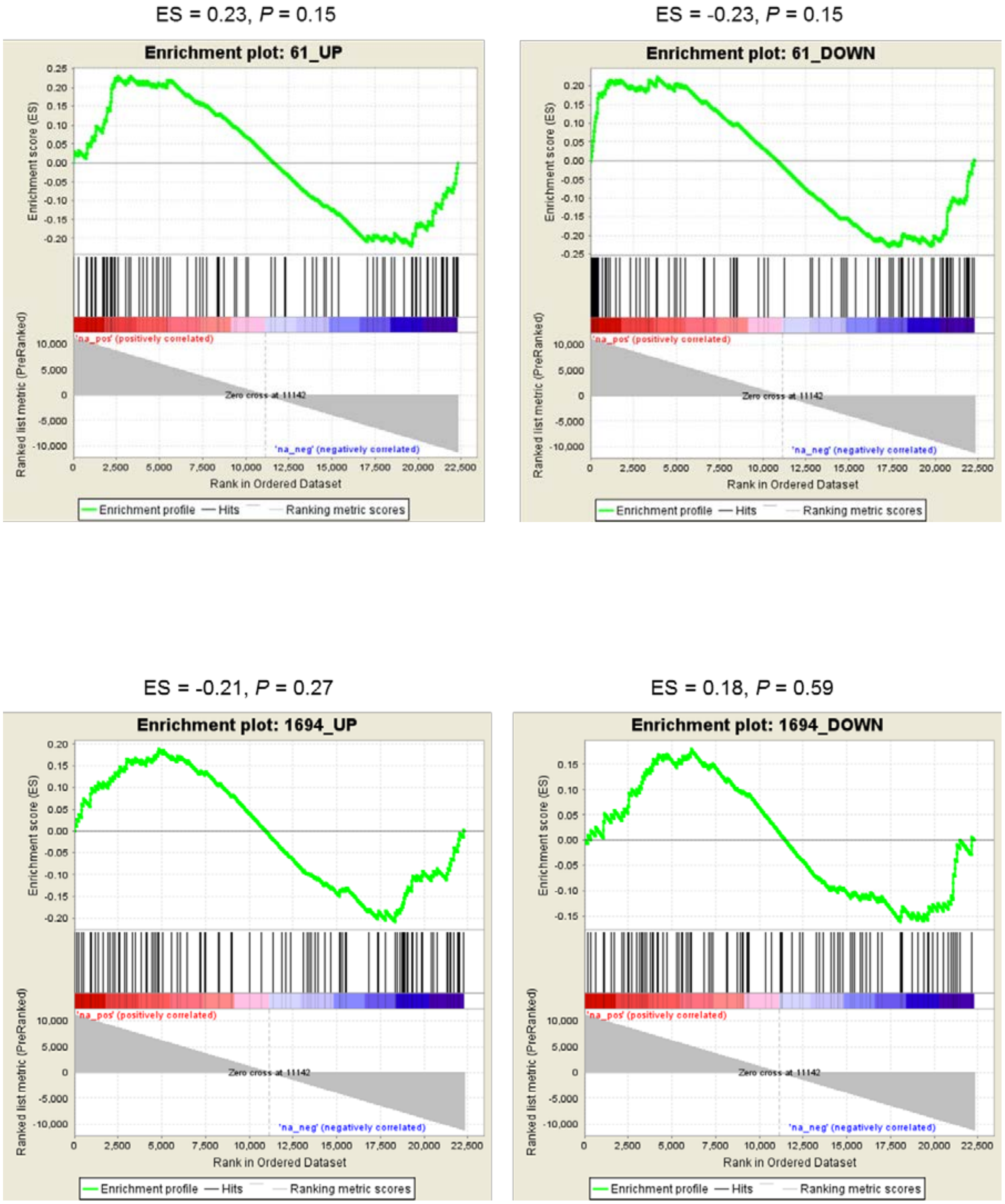

ES = 0.17, *P* = 0.67

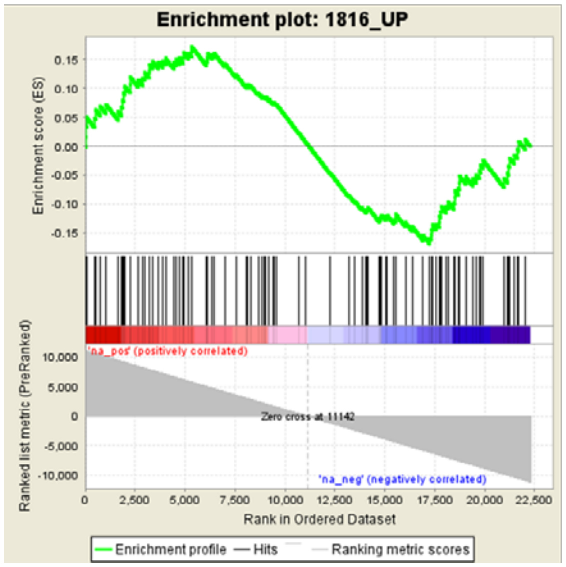

ES = 0.19, *P* = 0.52

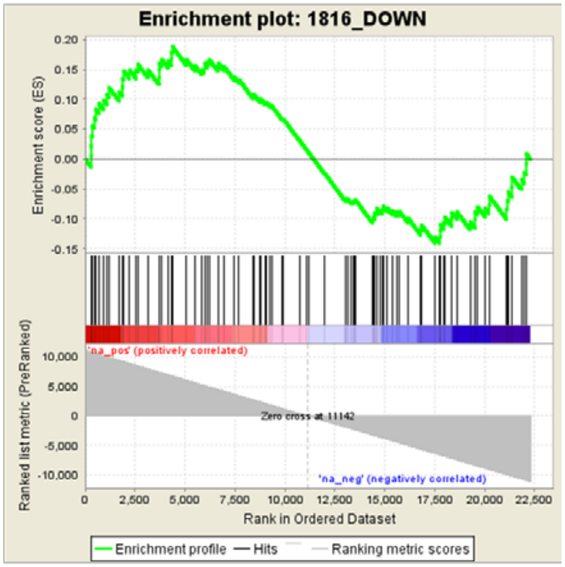

ES = -0.17, *P* = 0.69

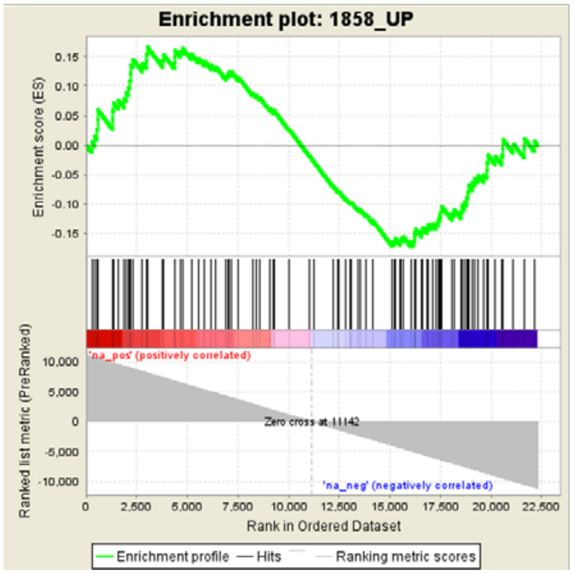

ES = 0.20, *P* = 0.35

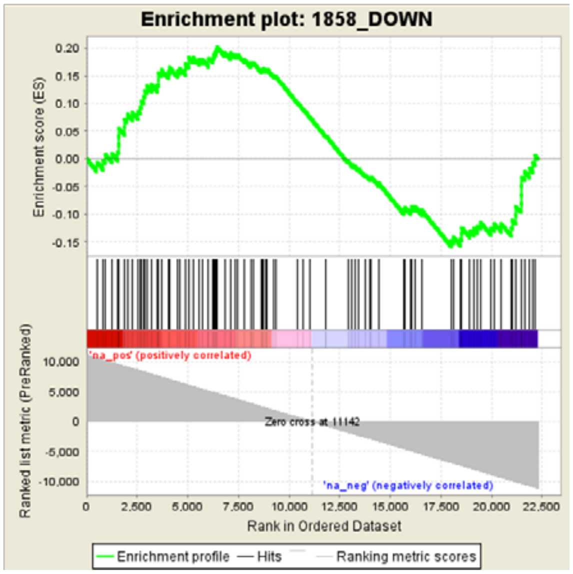

ES = -0.19, *P* = 0.42

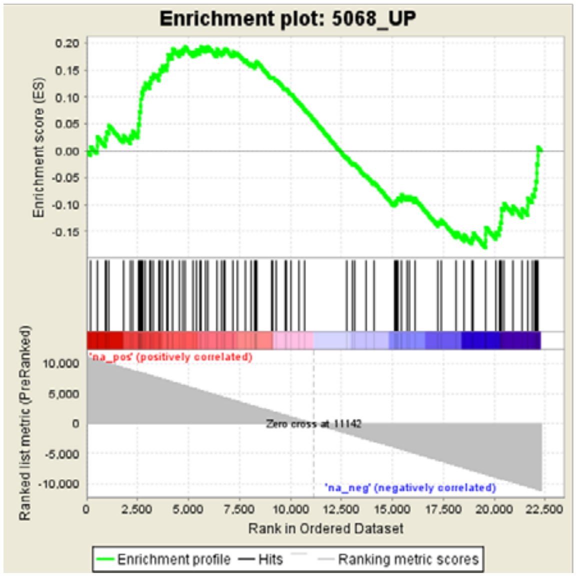

ES = 0.16, *P* = 0.78

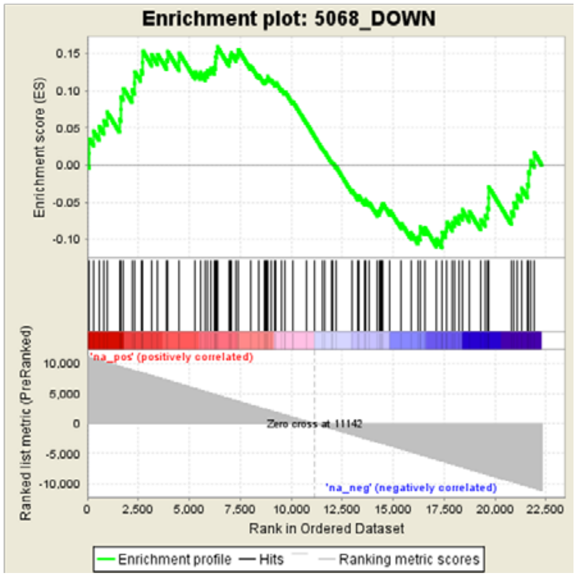

ES = 0.19, *P* = 0.51

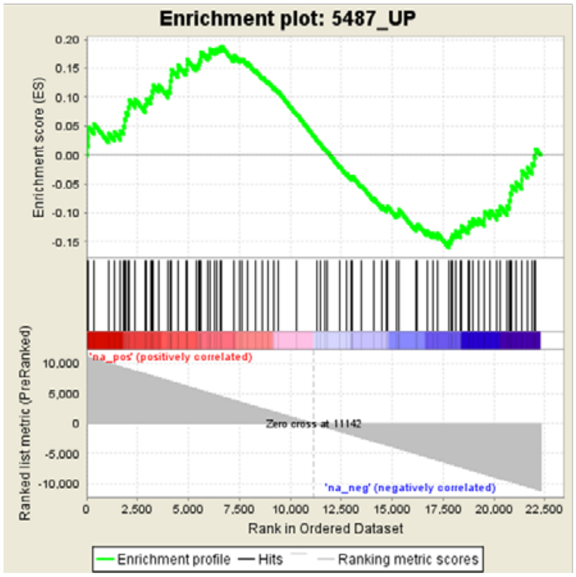

ES = -0.18, *P* = 0.54

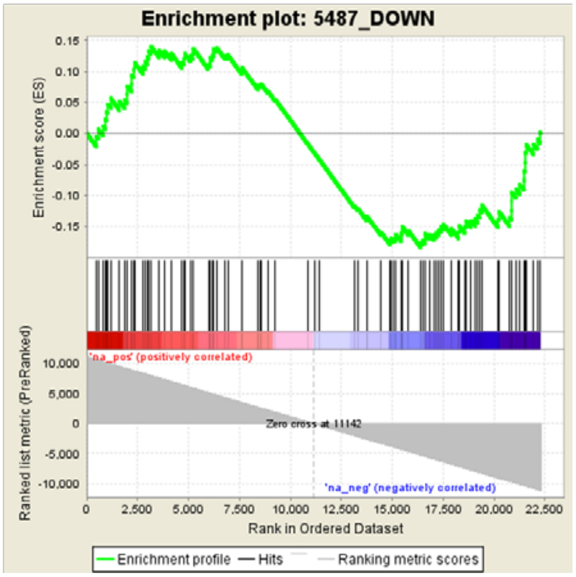

Supplement: S2 Fig — In each graph, the vertical black lines indicate the position of each of the probes of the studied probe set in the ordered, non-redundant data set. The green curve corresponds to the ES (enrichment score) curve, which is the running sum of the weighted enrichment score in GSEA. (PDF) [file pcbi.1004202.s002.pdf]
