# Supplementary material for: Deciphering Signaling Pathway Networks to Understand the Molecular Mechanisms of Metformin Action
Source: PLoS Comput Biol. 2015 Jun 17;11(6):e1004202. doi: 10.1371/journal.pcbi.1004202 (PMC4470683; doi:10.1371/journal.pcbi.1004202)
Supplement: S1 Table — (DOCX) [file pcbi.1004202.s013.docx]

**S1 Table Summary of data sources, software, and evaluation data used in the study**

|  | **Link** | **Purpose** |
| --- | --- | --- |
| ***Data sources*** |  |  |
| DrugBank | http://www.drugbank.ca/ | Collect drug-related genes |
| PharmGKB | https://www.pharmgkb.org/ | Collect drug-related genes |
| PubMed | http://www.ncbi.nlm.nih.gov/pubmed | Collect drug-related genes |
| Connectivity Map | https://www.broadinstitute.org/cmap/ | Infer drug-related TFs |
| Pathway Commons | http://www.pathwaycommons.org/about/ | Construct Signaling pathway network |
| TRANSFAC | http://www.biobase-international.com/product/transcription-factor-binding-sites | Construct signaling pathway network and infer drug-related TFs |
| ***Software and tools*** |  |  |
| Netwalker | http://bioinfo.vanderbilt.edu/netwalker/ | Network longitudinal movement |
| GenRev | http://bioinfo.mc.vanderbilt.edu/GenRev.html | Network lateral movement |
| GSEA | http://www.broadinstitute.org/gsea/index.jsp | Gene expression enrichment analysis |
| Ingenuity | http://www.ingenuity.com/ | Map probes to genes; |
| WebGestalt | http://bioinfo.vanderbilt.edu/webgestalt/ | Pathway enrichment analysis |
| CFinder | http://www.cfinder.org/ | Network module analysis |
| Cytoscape | http://www.cytoscape.org/ | Network visualization |
| ***Network disease gene evaluation data*** | | |
| GWAS catalog | http://www.genome.gov/gwastudies/ | T2D disease genes |
| Cancer gene census | http://cancer.sanger.ac.uk/cancergenome/projects/census/ | Cancer genes |
| T2D GWAS data | http://www.wtccc.org.uk/ | T2D associated genes based on GWAS data |
| Breast cancer GWAS data | http://dceg.cancer.gov/research/how-we-study/genomic-studies/cgems-summary | Breast cancer associated genes based on GWAS data |
| Pancreatic cancer GWAS data | http://dceg.cancer.gov/research/how-we-study/genomic-studies/cgems-summary | Pancreatic cancer associated genes based on GWAS data |
| Prostate cancer GWAS data | http://dceg.cancer.gov/research/how-we-study/genomic-studies/cgems-summary | Prostate cancer associated genes based on GWAS data |
| Metformin GWAS data |  | Metformin action related gene enrichment evaluation |
