# Supplementary material for: Deciphering Signaling Pathway Networks to Understand the Molecular Mechanisms of Metformin Action
Source: PLoS Comput Biol. 2015 Jun 17;11(6):e1004202. doi: 10.1371/journal.pcbi.1004202 (PMC4470683; doi:10.1371/journal.pcbi.1004202)
Supplement: S2 Table — (DOCX) [file pcbi.1004202.s014.docx]

**S2 Table Metformin upstream genes and their resources**

| **Gene symbol** | **Gene ID** | **DrugBank** | **PharmGKB** | **Text mining (PubMed ID)** |
| --- | --- | --- | --- | --- |
| *ABCC8* | 6833 |  |  | 12118200 |
| *ACACA* | 31 |  | Pharmacodynamics |  |
| *ACACB* | 32 |  | Pharmacodynamics |  |
| *ADIPOQ* | 9370 |  |  | 19622782, 16118250, 18997670 |
| *ATM* | 472 |  | Pharmacodynamics |  |
| *BDNF* | 627 |  | PGxResearch |  |
| *C11orf65* | 160140 |  | PGxResearch |  |
| *CAPN10* | 11132 |  |  | 19418728 |
| *CRTC2* | 200186 |  | Pharmacodynamics |  |
| *CYP3A4* | 1576 |  | Pharmacodynamics |  |
| *GHRL* | 51738 |  |  | 12364442 |
| *GPAM* | 57678 |  | Pharmacodynamics |  |
| *HMGCR* | 3156 |  | Pharmacodynamics |  |
| *IRS1* | 3667 |  |  | 19418728 |
| *KCNJ11* | 3767 |  | PGxResearch |  |
| *LEP* | 3952 |  |  | 18839053 |
| *LPL* | 4023 |  |  | 17374417 |
| *MAPK7* | 5598 |  |  | 19414528 |
| *MLXIPL* | 51085 |  | Pharmacodynamics |  |
| *MLYCD* | 23417 |  | Pharmacodynamics |  |
| *MTOR* | 2475 |  |  | 16354680 |
| *NAMPT* | 10135 |  |  | 17090638 |
| *NDUFA1* | 4694 |  | Pharmacodynamics |  |
| *NDUFA11* | 126328 |  | Pharmacodynamics |  |
| *NDUFA2* | 4695 |  | Pharmacodynamics |  |
| *NDUFAB1* | 4706 |  | Pharmacodynamics |  |
| *NDUFB4* | 4710 |  | Pharmacodynamics |  |
| *NDUFS1* | 4719 |  | Pharmacodynamics |  |
| *NDUFS2* | 4720 |  | Pharmacodynamics |  |
| *NDUFS4* | 4724 |  | Pharmacodynamics |  |
| *NDUFS6* | 4726 |  | Pharmacodynamics |  |
| *NDUFV1* | 4723 |  | Pharmacodynamics |  |
| *NR0B2* | 8431 |  |  | 17909097 |
| *NR1I2* | 8856 |  | Pharmacodynamics |  |
| *PPARG* | 5468 |  | PGxResearch | 19418728 |
| *PPARGC1A* | 10891 |  | Pharmacodynamics | 15864539 |
| *PRKAA1* | 5562 |  | Pharmacodynamics | 12890675 |
| *PRKAA2* | 5563 |  | Pharmacodynamics | 15371448 |
| *PRKAB1* | 5564 | Target | Pharmacodynamics |  |
| *PRKAB2* | 5565 |  | Pharmacodynamics |  |
| *PRKAG1* | 5571 |  | Pharmacodynamics |  |
| *PRKAG2* | 51422 |  | Pharmacodynamics |  |
| *PRKAG3* | 53632 |  | Pharmacodynamics |  |
| *RETN* | 56729 |  |  | 18446452 |
| *SERPINA12* | 145264 |  |  | 18375437 |
| *SIRT1* | 23411 |  | Pharmacodynamics |  |
| *SLC22A1* | 6580 | Transporter | PGxResearch, Pharmacokinemics | 17111267, 17701831, 17609683, 17476361, 19591196, 19336679 |
| *SLC22A2* | 6582 | Transporter | Pharmacokinemics | 17111267, 18401339 |
| *SLC22A3* | 6581 |  | PGxResearch, Pharmacokinemics | 19591196 |
| *SLC27A1* | 376497 | Transporter | Pharmacokinemics |  |
| *SLC27A2* | 11001 |  | Pharmacokinemics |  |
| *SLC29A4* | 222962 |  | Pharmacokinemics |  |
| *SLC2A4* | 6517 |  | Pharmacodynamics | 15864539 |
| *SLC30A8* | 169026 |  | PGxResearch |  |
| *SLC47A1* | 55244 |  | Pharmacokinemics | 19228809 |
| *SLC47A2* | 146802 |  | PGxResearch |  |
| *SLCO1B1* | 10599 |  |  | 18314419 |
| *SLCO1B3* | 28234 |  |  | 18314419 |
| *SREBF1* | 6720 |  | Pharmacodynamics |  |
| *SRR* | 63826 |  | PGxResearch |  |
| *STK11* | 6794 |  | Pharmacodynamics | 18000088 |
| *TBC1D4* | 9882 |  |  | 18771725 |
| *THBS1* | 7057 |  |  | 19414528, 18057090 |
| *TP53* | 7157 |  |  | 17638885 |
| *TP73* | 7161 |  |  | 18678646 |
