# Supplementary material for: Deciphering Signaling Pathway Networks to Understand the Molecular Mechanisms of Metformin Action
Source: PLoS Comput Biol. 2015 Jun 17;11(6):e1004202. doi: 10.1371/journal.pcbi.1004202 (PMC4470683; doi:10.1371/journal.pcbi.1004202)
Supplement: S3 Table — (DOCX) [file pcbi.1004202.s015.docx]

**S3 Table Metformin treatments from Connectivity Map database**

| **Instance ID** | **Batch ID** | **Concentration (M)** | **Duration (h)** | **Cell^a^** | **Array** |
| --- | --- | --- | --- | --- | --- |
| 1 | 1 | 0.00001 | 6 | MCF7 | HG-U133A |
| 2 | 1 | 0.00001 | 6 | MCF7 | HG-U133A |
| 3 | 1 | 0.0000001 | 6 | MCF7 | HG-U133A |
| 4 | 1 | 0.001 | 6 | MCF7 | HG-U133A |
| 1694 | 627 | 0.0000242 | 6 | MCF7 | HT_HG-U133A |
| 1816 | 628 | 0.0000242 | 6 | PC3 | HT_HG-U133A |
| 1858 | 629 | 0.0000242 | 6 | HL60 | HT_HG-U133A |
| 5068 | 718 | 0.0000242 | 6 | PC3 | HT_HG-U133A |
| 5487 | 737 | 0.0000242 | 6 | MCF7 | HT_HG-U133A |
| 61 | 2a | 0.00001 | 6 | MCF7 | HG-U133A |

| MCF7: human breast epithelial adenocarcinoma cell line derived from pleural effusion (ATCC# HTB-22) cultured in DMEM supplemented with 10% fetal bovine serum and 1% penicillin-streptomycin-glutamine  PC3: epithelial cell line established from human prostate adenocarcinoma (ATCC# CRL-1435) cultured in RPMI supplemented with 10% fetal bovine serum, 1% sodium pyruvate and 1% penicillin-streptomycin-glutamate  SKMEL5: human malignant melanoma cell line derived from a metastatic axillary node (ATCC# HTB-70) cultured in DMEM supplemented with 10% fetal bovine serum, 1% penicillin-streptomycin-glutamate, 1% non-essential amino acids and 1% sodium pyruvate  HL60: human promyelocytic cell line established by leukopheresis from promyelocytic leukemia (ATCC# CCL-240) cultured in RPMI supplemented with 10% fetal bovine serum and 1% penicillin-streptomycin-glutamate |
| --- |
