# Supplementary material for: Deciphering Signaling Pathway Networks to Understand the Molecular Mechanisms of Metformin Action
Source: PLoS Comput Biol. 2015 Jun 17;11(6):e1004202. doi: 10.1371/journal.pcbi.1004202 (PMC4470683; doi:10.1371/journal.pcbi.1004202)
Supplement: S4 Table — (DOCX) [file pcbi.1004202.s016.docx]

**S4 Table Metformin downstream genes encoding transcription factors inferred from metformin-induced gene expression data from Connectivity Map**

| **Gene symbol** | **Gene ID** | **Name** |
| --- | --- | --- |
| ATF1 | 466 | activating transcription factor 1 |
| ATF3 | 467 | activating transcription factor 3 |
| ATF4 | 468 | activating transcription factor 4 (tax-responsive enhancer element B67) |
| ATF7 | 11016 | activating transcription factor 7 |
| BACH1 | 571 | BTB and CNC homology 1, basic leucine zipper transcription factor 1 |
| EBF1 | 1879 | early B-cell factor 1 |
| ESR1 | 2099 | estrogen receptor 1 |
| ETS1 | 2113 | v-ets erythroblastosis virus E26 oncogene homolog 1 (avian) |
| FOS | 2353 | FBJ murine osteosarcoma viral oncogene homolog |
| FOSB | 2354 | FBJ murine osteosarcoma viral oncogene homolog B |
| FOSL1 | 8061 | FOS-like antigen 1 |
| FOSL2 | 2355 | FOS-like antigen 2 |
| FOXO1 | 2308 | forkhead box O1 |
| FOXO4 | 4303 | forkhead box O4 |
| GATA4 | 2626 | GATA binding protein 4 |
| HNF4A | 3172 | hepatocyte nuclear factor 4, alpha |
| HNF4G | 3174 | hepatocyte nuclear factor 4, gamma |
| HOXA5 | 3202 | homeobox A5 |
| HSF1 | 3297 | heat shock transcription factor 1 |
| HSF2 | 3298 | heat shock transcription factor 2 |
| IKZF1 | 10320 | IKAROS family zinc finger 1 (Ikaros) |
| IRF2 | 3660 | interferon regulatory factor 2 |
| IRF3 | 3661 | interferon regulatory factor 3 |
| IRF4 | 3662 | interferon regulatory factor 4 |
| IRF5 | 3663 | interferon regulatory factor 5 |
| IRF6 | 3664 | interferon regulatory factor 6 |
| IRF8 | 3394 | interferon regulatory factor 8 |
| JUN | 3725 | jun proto-oncogene |
| JUNB | 3726 | jun B proto-oncogene |
| JUND | 3727 | jun D proto-oncogene |
| MECOM | 2122 | MDS1 and EVI1 complex locus |
| MEF2A | 4205 | myocyte enhancer factor 2A |
| MEF2BNB-MEF2B | 4207 | MEF2BNB-MEF2B readthrough |
| MTF1 | 4520 | metal-regulatory transcription factor 1 |
| MYCN | 4613 | v-myc myelocytomatosis viral related oncogene, neuroblastoma derived (avian) |
| NR1H2 | 7376 | nuclear receptor subfamily 1, group H, member 2 |
| NR1H3 | 10062 | nuclear receptor subfamily 1, group H, member 3 |
| NR1I2 | 8856 | nuclear receptor subfamily 1, group I, member 2 |
| NR1I3 | 9970 | nuclear receptor subfamily 1, group I, member 3 |
| NR2F1 | 7025 | nuclear receptor subfamily 2, group F, member 1 |
| NR2F2 | 7026 | nuclear receptor subfamily 2, group F, member 2 |
| NR3C1 | 2908 | nuclear receptor subfamily 3, group C, member 1 (glucocorticoid receptor) |
| PATZ1 | 23598 | POZ (BTB) and AT hook containing zinc finger 1 |
| POU3F1 | 5453 | POU class 3 homeobox 1 |
| PPARA | 5465 | peroxisome proliferator-activated receptor alpha |
| PPARD | 5467 | peroxisome proliferator-activated receptor delta |
| PPARG | 5468 | peroxisome proliferator-activated receptor gamma |
| PRDM1 | 639 | PR domain containing 1, with ZNF domain |
| RARA | 5914 | retinoic acid receptor, alpha |
| RARB | 5915 | retinoic acid receptor, beta |
| RARG | 5916 | retinoic acid receptor, gamma |
| REL | 5966 | v-rel reticuloendotheliosis viral oncogene homolog (avian) |
| RELA | 5970 | v-rel reticuloendotheliosis viral oncogene homolog A (avian) |
| RREB1 | 6239 | ras responsive element binding protein 1 |
| RXRA | 6256 | retinoid X receptor, alpha |
| RXRB | 6257 | retinoid X receptor, beta |
| SMAD3 | 4088 | SMAD family member 3 |
| SRF | 6722 | serum response factor (c-fos serum response element-binding transcription factor) |
| TBP | 6908 | TATA box binding protein |
| TBX3 | 6926 | T-box 3 |
| TCF7 | 6932 | transcription factor 7 (T-cell specific, HMG-box) |
| TCF7L1 | 83439 | transcription factor 7-like 1 (T-cell specific, HMG-box) |
| TEAD3 | 7005 | TEA domain family member 3 |
| TEAD4 | 7004 | TEA domain family member 4 |
| TFAP2C | 7022 | transcription factor AP-2 gamma (activating enhancer binding protein 2 gamma) |
| ZIC3 | 7547 | Zic family member 3 |

Down

AR 12 421 12258 197 0.017221543653562 1

ATF1 13 389 12290 197 0.00474238844796873 1

ATF3 11 376 12303 197 0.0184534066432999 1

ATF4 10 392 12287 197 0.0428733536214425 1

CREM 10 398 12281 197 0.0455984669323195 1

DEAF1 1 1 12678 197 0.0155375029576465 1

EBF1 7 204 12475 197 0.0257438960147878 1

EGR4 7 200 12479 197 0.0237801982090403 1

ELF2 1 338 12341 197 0.0256422332313362 1

FOXN1 8 192 12487 197 0.00718552303397305 1

FOXO1 10 315 12364 197 0.0151790294733455 1

FOXO4 10 314 12365 197 0.0149228385635728 1

GATA4 11 347 12332 197 0.0117178049646702 1

HNF4A 20 791 11888 197 0.0101745401280692 1

HNF4G 13 431 12248 197 0.00972535205994212 1

IKZF1 16 634 12045 197 0.0182949087325125 1

MTF1 7 200 12479 197 0.0237801982090403 1

NR1H2 8 195 12484 197 0.0077884903165688 1

NR1H3 8 195 12484 197 0.0077884903165688 1

NR1I2 13 469 12210 197 0.0166436788837227 1

NR1I3 13 471 12208 197 0.0170764244862122 1

NR2F1 22 712 11967 197 0.000912071663588099 0.253555922477491

NR2F2 23 729 11950 197 0.000549190403197581 0.152674932088928

PATZ1 6 172 12507 197 0.0339560160250583 1

RARA 11 381 12298 197 0.0198129628511303 1

RARB 11 375 12304 197 0.0181885025745932 1

RARG 11 376 12303 197 0.0184534066432999 1

RXRA 14 560 12119 197 0.0258143581291124 1

RXRB 13 463 12216 197 0.015387126875936 1

SP1 23 967 11712 197 0.0116988265054841 1

SP3 16 711 11968 197 0.0363006882881405 1

SREBF1 3 497 12182 197 0.0316568879822067 1

TBX3 1 1 12678 197 0.0155375029576465 1

TCF7L2 6 192 12487 197 0.0487313489972601 1

TEAD1 6 180 12499 197 0.0395929146440593 1

TEAD3 1 3 12676 197 0.0451823486733181 1

TEAD4 2 4 12675 197 0.0013972452413964 0.388434177108198

TFAP2C 15 602 12077 197 0.0228530854306518 1

Up
