# Supplementary material for: Deciphering Signaling Pathway Networks to Understand the Molecular Mechanisms of Metformin Action
Source: PLoS Comput Biol. 2015 Jun 17;11(6):e1004202. doi: 10.1371/journal.pcbi.1004202 (PMC4470683; doi:10.1371/journal.pcbi.1004202)
Supplement: S10 Table — (DOCX) [file pcbi.1004202.s022.docx]

**S10 Table Summary of three cancer GWAS data**

| **GWAS** | **dbGaP ID** | **Cases** | **Controls** | **Number of genes^a^** | **Number of genes with smallest *P*< 0.05** |
| --- | --- | --- | --- | --- | --- |
| Breast cancer | phs000147.v1.p1 | 1145 | 1142 | 19542 | 5976 |
| Pancreatic cancer | phs000206.v1.p1 | 1924 | 2042 | 19574 | 6475 |
| Prostate cancer | phs000207.v1.p1 | 1146 | 1097 | 19615 | 6348 |

^a^ For the three GWAS data sets, each number denotes the number of genes with genotyping data in corresponding GWAS data.
